# The Histone H3K27 Methylation Mark Regulates Intestinal Epithelial Cell Density-Dependent Proliferation and the Inflammatory Response

**DOI:** 10.1002/jcb.24463

**Published:** 2012-11-28

**Authors:** Naomie Turgeon, Mylène Blais, Jean-François Delabre, Claude Asselin

**Affiliations:** Faculté de médecine et des sciences de la santé, Département d'anatomie et biologie cellulaire, Pavillon de recherche appliquée sur le cancer, Université de SherbrookeSherbrooke, Québec, Canada J1E 4K8

**Keywords:** H3K27, IEC-6, INFLAMMATION, PROLIFERATION, INTESTINAL EPITHELIAL CELL, STAT3, P38, Suz12

## Abstract

Polycomb-group proteins form multimeric protein complexes involved in transcriptional silencing. The Polycomb Repressive complex 2 (PRC2) contains the Suppressor of Zeste-12 protein (Suz12) and the histone methyltransferase Enhancer of Zeste protein-2 (Ezh2). This complex, catalyzing the di- and tri-methylation of histone H3 lysine 27, is essential for embryonic development and stem cell renewal. However, the role of Polycomb-group protein complexes in the control of the intestinal epithelial cell (IEC) phenotype is not known. We show that Suz12 and Ezh2 were differentially expressed along the intestinal crypt-villus axis. ShRNA-mediated Suz12 depletion in the IEC-6 rat crypt-derived cell line decreased Ezh2 expression and H3K27 di-trimethylation. Suz12-depleted cells achieved higher cell densities after confluence, with increased cyclin D2 and cyclin D3 protein levels, and increased STAT3 activation in post-confluent cells. Suz12 depletion specifically increased mostly developmental, cell adhesion and immune response gene expression, including neuronal and inflammatory genes. Suz12 depletion directly and indirectly de-regulated the IL-1β-dependent inflammatory response, as demonstrated by decreased MAPK p38 activation as opposed to JNK activation, and altered basal and stimulated expression of inflammatory genes, including transcription factors such as C/EBPβ. Of note, this positive effect on cell proliferation and inflammatory gene expression was revealed in the absence of the cyclin-dependent kinase inhibitor p16, a main target negatively regulated by PRC2. These results demonstrate that the PRC2 complex, in addition to keeping in check non-IEC differentiation pathways, insures the proper IEC response to cell density as well as to external growth and inflammatory signals, by controlling specific signaling pathways. J. Cell. Biochem. 114: 1203–1215, 2013. © 2012 Wiley Periodicals, Inc.

Rapid intestinal epithelial cell (IEC) turnover is regulated and maintained by a population of intestinal crypt stem cells, which give rise to all IEC lineages [Scoville et al., 2008]. Differentiated enteroendocrine cells, enterocytes, and goblet mucus-producing cells reside in intestinal villi, as opposed to Paneth cells which occupy the crypt compartment and secrete antimicrobial peptides. While many different molecular pathways regulate the differentiation or maintenance of the undifferentiated state of stem cells, including IEC stem cells [Van der Flier and Clevers, 2009], the extent of epigenetic-dependent transcriptional mechanisms in the control of IEC proliferation and differentiation remains to be determined.

Polycomb Group (PcG) proteins are one class of negative epigenetic transcriptional regulators controlling among others, the expression of homeotic (Hox) genes in *Drosophila* and in mammals, by maintaining their repressive transcriptional state [Simon and Kingston, 2009]. Genetic studies demonstrate that PcG protein mutations lead to inappropriate Hox gene expression, resulting in flies with transformed body segments. PcG containing complexes silence gene expression through the formation of multiprotein complexes remodeling as well as compacting chromatin, leading to transcriptional inhibition of genes associated with these regions [Sauvageau and Sauvageau, 2010]. In mammals, PcG complexes include the Polycomb repressive complexes (PRC) PRC1 and PRC2. Of these, PRC2 is composed, among others, of the histone methyltransferase Enhancer of Zeste protein-2 (Ezh2), the Embryonic Ectoderm Development protein (Eed), the Suppressor of Zeste-12 protein (Suz12), and the histone-binding proteins RbAp46/48 [Jones and Wang, 2010; Margueron and Reinberg, 2011]. When recruited to chromatin, the PRC2 Ezh2 methyltransferase di- and tri-methylates histone H3 on lysine 27 (H3K27me2 and H3K27me3). This histone modification is recognized by the PRC1 complex, which establishes PcG-mediated repression. Genome-wide localization studies in embryonic stem cells have uncovered H3K27 methyl mark enrichment in many developmental regulator genes, including Hox genes. In addition, both the negative H3K27me3 mark and the positive H3K4me3 mark are present in bivalent domains found in genes regulated during embryonic stem cell differentiation [Bernstein et al., 2006; Mikkelsen et al., 2007]. PRC2 subunits are essential for embryonic development and stem cell differentiation, as shown by Ezh2 depletion [O'Carroll et al., 2001; Pasini et al., 2004]. The Ezh2 methyltransferase controls skeletal muscle differentiation [Caretti et al., 2004], B cell development [Su et al., 2003], pancreatic β-cell regeneration [Chen et al., 2009a] and epidermal differentiation [Ezhkova et al., 2009]. Suz12 is required for embryonic stem cell differentiation, PRC2 histone methyltransferase activity, and Ezh2 stability [Cao and Zhang, 2004; Pasini et al., 2004; Pasini et al., 2007]. While PRC2 plays a major role in maintaining stem-cell pluripotency [Jones and Wang, 2010], PRC2 inactivation leads to cell-specific differentiation defects [Prezioso and Orlando, 2011], such as increased myoblast differentiation [Caretti et al., 2004] or decreased adipocyte differentiation [Wang et al., 2010]. In addition to their cell fate regulatory functions, PcG proteins regulate cell proliferation, notably through repression of the cyclin-dependent kinase inhibitor p16 locus [Maertens et al., 2009]. While PRC2 protein levels are increased in a number of cancers, the identification of various mutations altering PcG protein functions suggests both tumor suppressive as well as oncogenic roles [Sauvageau and Sauvageau, 2010].

However, the exact role of PRC2 and the H3K27 methyl mark in the control of IEC gene expression is not known. We addressed the role of PRC2 by downregulating one component of the PRC2 complex, namely Suz12, in the IEC-6 rat intestinal epithelial cell line derived from intestinal crypt cells of weaned rats [Quaroni and May, 1980]. We show that Suz12 depletion in IEC-6 cells deregulates differentiation-specific genes, and alters density-dependent cell proliferation as well as cell-intrinsic inflammatory responses through specific signaling pathways. These results demonstrate that the PRC2 complex, in addition to keeping in check non-IEC differentiation pathways, insures the proper IEC response to cell density as well as to external growth and inflammatory signals.

## MATERIAL AND METHODS

### Cell Culture

The rat intestinal epithelial cell line IEC-6, derived from 18- to 24-day-old rat small intestine, displays an undifferentiated small intestinal crypt cell phenotype, a normal karyotype and is not tumorigenic [Quaroni and May, 1980]. Cells were grown in Dulbecco's modified Eagle medium (DMEM) with 5% fetal bovine serum (FBS). When needed, cells at 80% confluence were stimulated with IL-1β (10 ng/ml; BioShop Canada, Inc., Burlington, ON).

### Retroviral Infection

Multiple shRNA lentiviral constructs were tested in IEC-6 cells for their ability to down-regulate Suz12 expression (MISSION shRNA lentiviral transduction particles, Sigma–Aldrich Canada, Oakville, ON). Forty percent confluent cells were infected in medium supplemented with 4 µg/ml polybrene (Sigma–Aldrich Canada) for 6 h. Two days after infection, cell populations were selected with 2 µg/ml puromycin (Sigma–Aldrich Canada). The clone TRCN0000038728 was selected: the shRNA against Suz12 (GCTGACAATCAAATGAATCAT) is conserved in rat (accession number FM084383), murine (accession number NM_199196) and human (accession number NM_015355.2) Suz12 sequences. Efficiency of infection was estimated at 25%.

### Cell Growth Measurement

Ten thousand control or ShSuz12 IEC-6 cells were plated in each well. Cell growth was measured between 4 and 23 days with a cell particle counter (Z1 Series Coulter Counter, Beckman Coulter, Etobicoke, ON). Each experiment was done three times in triplicate. Results were analyzed by ANOVA, and were considered statistically significant with *P* ≤ 0.05. Results are representative of three independent experiments.

### Cell Fractionation Along the Crypt-Villus Axis

Murine jejunum and ileum segments inverted onto polyethylene tubing were washed extensively with KRP buffer, pH 7.5, as described previously [Boulanger et al., 2005]. Segments were incubated with ice-cold isolation buffer (2.5 mM EDTA and 0.25 mM NaCl), and cell suspension was recovered, at every 2 min interval, at 1,500 RPM for 5 min. After an ice-cold KRB buffer wash, pellets were lysed in chilled Triton lysis buffer (150 mM NaCl, 1 mM EDTA, 40 mM Tris, pH 7.6, 1% Triton X-100, 0.1 mM PMSF, 10 µg/ml leupeptin, 1 µg/ml pepstatin, 10 µg/ml aprotinin, 0.1 mM orthovanadate, and 40 mM β-glycerophosphate).

### Western Blot Analysis

Nuclear extracts were prepared as described previously [Gheorghiu et al., 2001]. Histones from control and ShSuz12 IEC-6 cells were isolated as described before [Désilets et al., 2000]. Nuclei resuspended in 0.1 ml H_2_SO_4_ 0.4 N were incubated at 4°C for 2 h. After overnight incubation at −20°C with 1 ml acetone, histones were resuspended in Laemmli buffer (62.5 mM Tris–HCl, pH 6.9, 2% SDS, 1% β-mercaptoethanol, 10% glycerol, and 0.04% bromophenol blue). Total cellular extracts from sub-confluent or 5-day confluent control or ShSuz12 IEC-6 cells were prepared as done before and diluted in Laemmli buffer [Gheorghiu et al., 2001]. To measure MAPK p38 (Mapk14) or JNK (Mapk8) activation, confluent control or ShSuz12 IEC-6 cells were induced with 10 ng/ml of IL-1β for 10 min, 30 min and 2 h. Protein concentrations were measured by the Bradford method (Bio-Rad Protein Assay kit, Bio-Rad Laboratories, Mississauga, ON) or BCA method (Pierce BCA Protein Assay Kit, Thermo Scientific, Rockford). Proteins were loaded on a 15% for histones, or a 10% SDS–polyacrylamide gel and electroblotted on a PVDF membrane (Roche Molecular Biochemicals, Laval, QC). Membranes were incubated with rabbit, mouse or goat polyclonal antibodies against Ezh2, Stat3, and phospho-Stat3 (Tyr705), NF-κB p65 (Rela) and phospho-NF-κB p65 (Ser536; New England Biolabs, Mississauga, ON); β-catenin (Ctnnb1; Abcam, Cambridge, MA); activated β-catenin (antibody recognizing dephosphorylated Ser37 or Thr41), Suz12, acetyl histone H3, acetyl histone H4, trimethyl-histone H3 (Lys27), phospho-histone H3 (Ser10), actin and Cyclin D3 (Ccnd3; Millipore, Billerica, MA); Pcna (Calbiochem, Cambridge, MA); CyclinD (Ccnd1, Ccnd2), laminB (Lmnb1), C/EBPβ and HoxB13 (Santa Cruz Biotechnology, Santa Cruz, CA); JNK (Mapk8), phospho-JNK (Thr183/Tyr185), p38 (Mapk14), and phospho-p38 (Thr180/Tyr182; New England Biolabs, Mississauga, ON) overnight at 4°C. Immune complexes were detected with the Amersham ECL™ Western Blotting detection reagents (GE Healthcare, Buckinghamshire, UK) according to the manufacturer's instructions. When needed, quantification of band intensity was performed with the Quantity One software (Bio-Rad Laboratories). Results are representative of two or three independent experiments.

### Microarray Analysis

Control or ShSuz12 IEC-6 cell total RNAs were isolated with the Rneasy kit (Qiagen, Mississauga, ON, Canada), according to the manufacturer's instructions. RNA samples from three independent experiments were used for microarray analysis. cDNA preparation, microarray assay and primary analysis were performed at the Microarray platform of the McGill University and Genome Quebec Innovation Centre, using standard Affimetrix protocols and subsequent hybridization on Affymetrix GeneChip Rat Genome RAE230 2.0 arrays representing over 28,000 rat genes. Data analysis, normalization average difference and expression measurements were performed using Flexarray software version 1.6.1. Background correction and normalization were done with the robust multi-array average (RMA) algorithm. Significant statistical differences were calculated using Student's *t*-test. The cut-off for statistical significance was set at a *P*-value of 0.05 or below. Gene ontology searches were performed using “Database for Annotation, Visualization, and Integrated Discovery, version 2008” and GOTERM_BP_ALL annotations (DAVID: http://david.abcc.ncifcrf.gov/) [Dennis et al., 2003] or with the Transcriptome, ontology, phenotype, proteome, and pharmacome annotations based gene list (ToppGene) suite (http://toppgene.cchmc.org/) [Chen et al., 2009b]. We also searched for molecular signatures by comparing our dataset to the collection of annotated gene sets MsigDB (http://www.broadinstitute.org/gsea/msigdb/index.jsp). The same parameters were used for all analysis.

### Semi-Quantitative RT-PCR Analysis

Total cellular RNAs from ShControl or ShSuz12 IEC-6 cells, treated with or without IL-1β for 4 or 24 h were prepared with the Rneasy kit (Qiagen). cDNAs were synthesized using oligo(dT) and Superscript II reverse transcriptase (Invitrogen, Frederick, MD), following the manufacturer's protocol. cDNA products were amplified with the Taq PCR Master Mix Kit (Qiagen) using PCR primers designed against the corresponding *Rattus norvegicus* cDNAs for achaete-scute complex homolog 1 (Ascl1), calcitonin receptor (Calcr), amiloride-sensitive cation channel 1 (Accn1), Necdin (Ndn), solute carrier family 6 (neutral amino acid transporter), member 15 (Slc6a15), ALX homeobox 1 (Cart1 or Alx1), UDP glycosyltransferase 8 (Ugt8), Hu antigen B (Elavl2), homeobox B13 (Hoxb13), insulin-like growth factor binding protein 5 (Igfbp5), SIX homeobox 3 (Six3), WNT inhibitory factor 1 (Wif1), dickkopf 2 (Dkk2), dual specificity protein phosphatase 2 (Dusp2), dual specificity protein phosphatase 8 (Dusp8), interleukin 6 (Il6) in order to generate 300–400 bp amplified products (Supplementary Table I; http://biotools.umassmed.edu/bioapps/primer3_www.cgi). Expression of cell cycle regulators, namely Ccnd1, Ccnd2, Cdkn1a (p21), Cdkn1b (p27), and Cdkn1c (p57), was also determined. To assess the IL-1β response in both cell lines, we selected IL-1β-induced targets we have identified in IL-1β induced IEC-6 cells, namely Ccl2, Ccl5, Cxcl1, Cxcl2, Cxcl10, and thiostatin (Kng1; Supplementary Table I). cDNA amplification was performed by a first 94°C cycle for 5 min, followed by 26 cycles of 1 min at 94°C, 1 min at 62°C, and 1 min at 72°C, and a final 10 min cycle at 72°C. Relative quantification was determined by glyceraldehyde-3 phosphate-dehydrogenase (Gapdh) amplification. Amplified PCR products were separated on a 1.4% agarose gel and visualized by ethidium bromide staining. Results are representative of two to three independent experiments.

### Chromatin Immunoprecipitation Assays

Chromatin immunoprecipitation (ChIP) assays were performed as previously described [Blais et al., 2007], with an EZ CHIP assay kit (Millipore). Control or ShSuz12 IEC-6 cells were crosslinked with 1% formaldehyde for 10 min at 37°C. Chromatin was immunoprecipitated with rabbit polyclonal antibodies against trimethyl histone H3 (Lys27) and dimethyl histone H3 (Lys4; Millipore). One percent of the lysate was used to verify the amount of DNA for each immunoprecipitation. Purified immunoprecipitated DNA was diluted 1:20 before amplification of Ncd (primers −230 to −211 (5′-TCCCCACTATCACCCATCTC-3′) and −35 to −16 (5′-GTGCGCTTTACTGAGCACTG-3′) rat promoter sequences. Amplified DNAs were separated and visualized on a 2% agarose gel. Experiments were performed three times independently.

### Cytokine Secretion Assay

To assess cytokine and chemokine protein expression, supernatants from confluent control and Suz12-depleted cells were recovered, diluted and mixed with biotinylated detection antibodies. The sample and antibody mixture was incubated with the Rat Cytokine Array Panel A membrane (Proteome Profiler Array, R&D Systems, Minneapolis, MN), allowing the detection among others, of Cxcl1, Cxcl2, Cxcl10, Ccl5, and Il6. Of note, the expression of these targets have also been evaluated by semi-quantitative RT-PCR. The immune complex was revealed by Streptavidin-HRP and chemiluminescent detection reagents. Fluorescence intensity was measured with a Microarray scanner.

## RESULTS

### Suz12 Is Expressed in a Crypt-to-Villus Gradient in the Murine Intestinal Epithelium

**Fig. 1:**
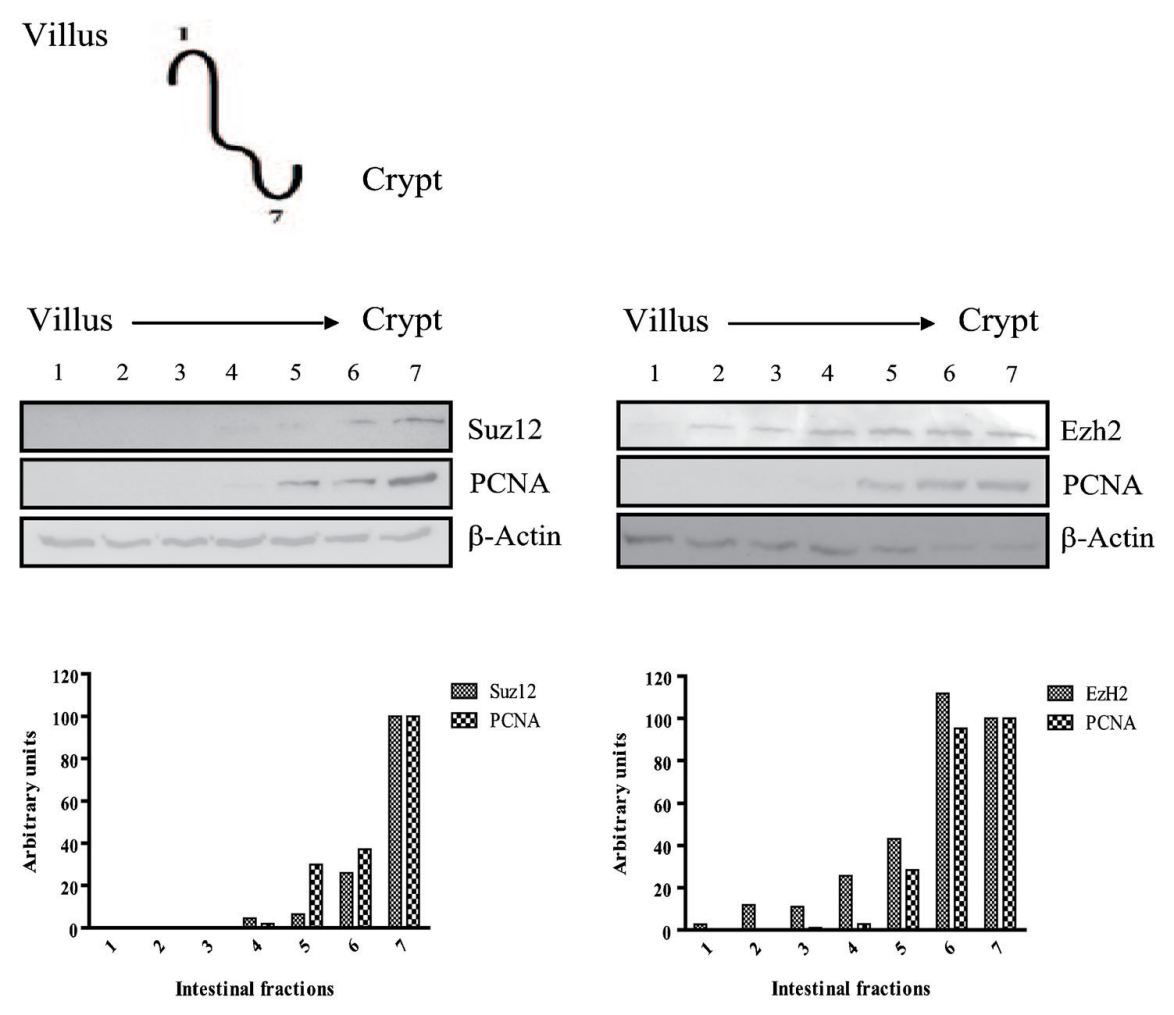
Suz12 and Ezh2 are differently expressed along the murine intestinal crypt-villus axis. Murine crypt and villus epithelial cell fractions (1–7) were analyzed for Suz12, Ezh2, PCNA, and actin expression by Western blotting. The villus to crypt gradient is indicated. The histograms indicate the ratio of Suz12, Ezh2 and PCNA band intensities normalized to β-actin. Relative quantification of band intensity was performed with the Quantity One software. Results are representative of two independent experiments.

We first determined the Suz12 and Ezh2 pattern of expression along the murine jejunum crypt-villus axis. Both Suz12 and histone methyltransferase Ezh2 proteins, as well as PCNA, a marker of cell proliferation, displayed a decreasing gradient of expression from the crypt to the villus, as determined by Western blot analysis of IEC crypt to villus protein fractions (Fig. 1). Thus, the PCR2 components Suz12 and Ezh2 are mostly expressed in non-differentiated proliferative crypt IEC.

### Suz12 Depletion in IEC-6 Cells Results in a Loss of H3K27 Trimethylation

**Fig. 2:**
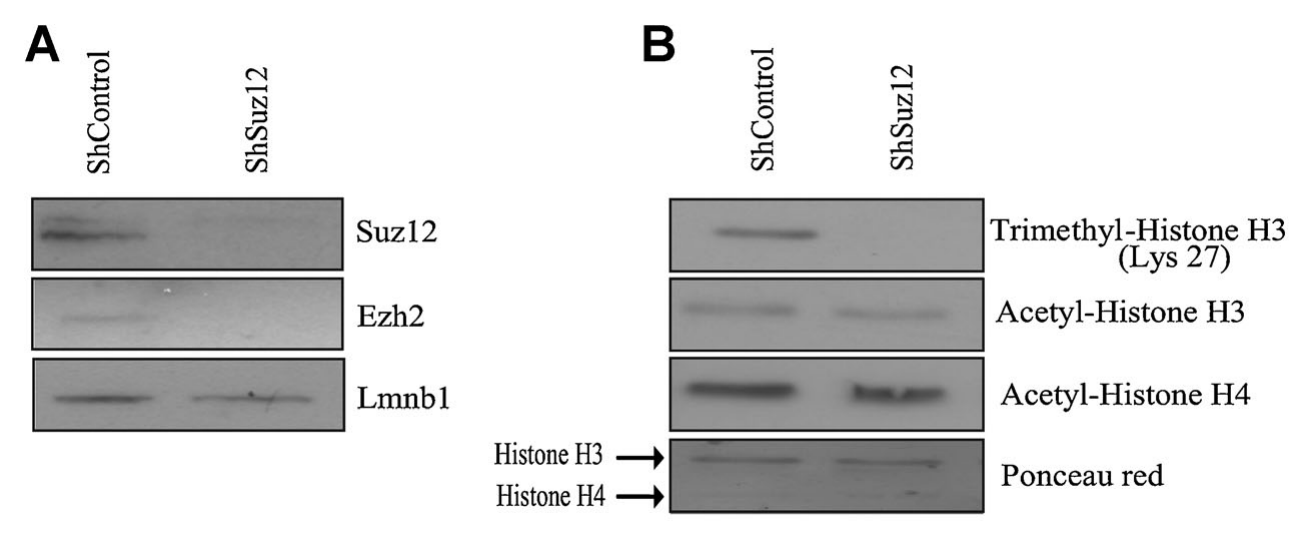
Suz12 depletion leads to Ezh2 and H3K27 trimethylation loss in intestinal epithelial cells. A: IEC-6 cells were stably infected with lentiviruses encoding a control short hairpin RNA (shRNA, scrambled sequence) or encoding a Suz12-specific shRNA. After puromycin selection, nuclear extracts were prepared and separated by SDS–PAGE. Suz12, Ezh2, and laminB (Lmnb1) expression was analyzed by Western blotting. B: ShControl and ShSuz12 IEC-6 cell histones were separated by SDS–PAGE. H3K27 trimethylation as well as H3 and H4 acetylation levels were assessed by Western blot with specific antibodies. Ponceau Red was used to stain the membranes in order to verify the amount of histones loaded. Results are representative of two independent experiments.

Suz12 is a component of the PRC2 complex containing the histone methyltransferase Ezh2 implicated in transcriptional silencing and differentiation [Margueron and Reinberg, 2011]. To investigate the role of Suz12 and the H3K27 methylation mark in IEC, we generated by retroviral infection an IEC-6 rat intestinal epithelial cell line expressing a ShRNA against Suz12. A control cell line was obtained by infection with a Sh scrambled vector. We observed by immunoblotting a decrease of both Suz12 and Ezh2 protein expression (Fig. 2A). This correlated with decreased global H3K27 trimethylation levels (Fig. 2B). In contrast, H3 and H4 acetylation (Fig. 2B), as well as H3K4, H3K9 and H3K36 dimethylation levels (data not shown), were not altered in Suz12-depleted cells. Thus, Suz12 depletion leads to reduced Ezh2 protein and global H3K27 methylation levels.

### Suz12 Depletion Alters IEC-6 Cell Density-Dependent Proliferation

**Fig. 3:**
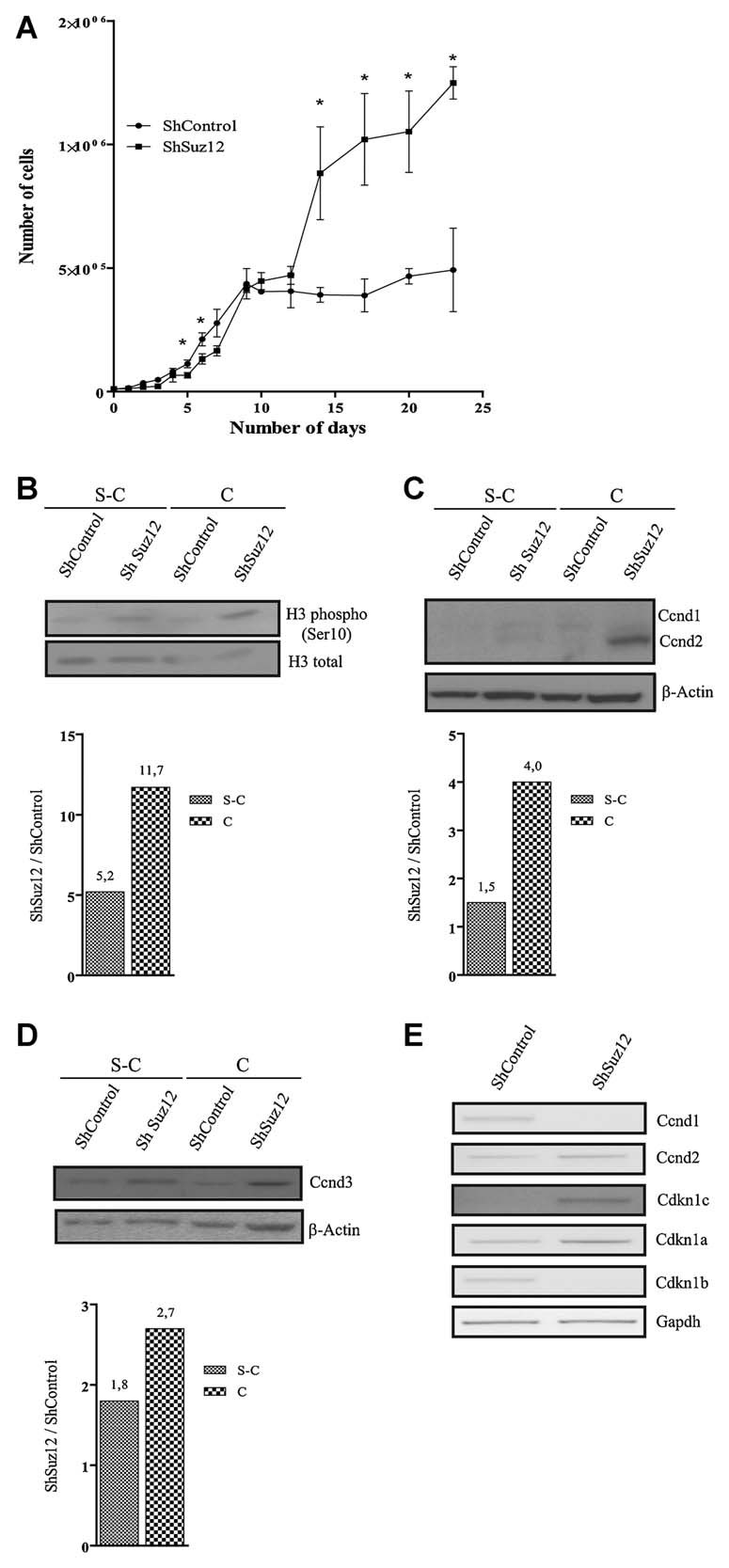
Suz12 depletion alters intestinal epithelial cell proliferation. Cell growth of ShControl and ShSuz12 IEC-6 cells was measured by cell counting between 4 and 23 days. Error bars represent the SEM of a representative experiment repeated more than three times, and done in triplicate (A). Twenty-five microgram of whole cell protein extracts from sub-confluent (s-c) or 5-day confluent (c) ShControl and ShSuz12 IEC-6 cells were separated by SDS–PAGE and transferred to PVDF membranes for Western blot analysis of histone H3 and phospho-histone H3 (Ser10; B), Cyclin D1 (Ccnd1), Cyclin D2 (Ccnd2), and actin as a loading control (C), Cyclin D3 (Ccnd3) and actin as a loading control (D). The histograms indicate the ratio of sub-confluent and confluent ShSuz12 versus ShControl band intensities normalized to β-actin. Relative quantification of band intensity was performed with the Quantity One software. Results are representative of two independent experiments. E: Total RNAs were isolated from confluent control or Suz12 depleted cells. Expression levels of Ccnd1, Ccnd2, p21 (Cdkn1a), p27 (Cdkn1b), and p57 (Cdkn1c) were verified by semi-quantitative RT-PCR, with Gapdh as a loading control. The amplified products were separated on 1.5% agarose gels. Results are representative of two independent experiments.

Suz12-depleted cells grew to higher cell densities after confluence, in contrast to control cells (Fig. 3A). In addition, ShSuz12 cell proliferation was increased in conditions of serum deprivation, as opposed to control cells, as determined by a MTT proliferation assay (data not shown). Western blot analysis showed an increase in H3Ser10 phosphorylation, a histone modification correlating with cell proliferation [Dong and Bode, 2006], along with increases of both Ccnd2 and Ccnd3 protein levels, in confluent Suz12-depleted cells, in contrast to a decrease in Ccnd1 levels (Fig. 3B–D). Indeed, while semi-quantitative RT-PCR analysis confirmed decreased Ccnd1 mRNA levels, Ccnd2 mRNAs were induced in Suz12-depleted cells (Fig. 3E). In addition, expression of cyclin-dependent kinase inhibitors was modified. While both Cdkn1a and Cdkn1c mRNA levels were increased, Cdkn1b expression was abrogated in Suz12-depleted cells (Fig. 3E). Of note, the p16 cyclin-dependent inhibitor Cdkn2a protein and mRNA were absent both in control as well as Suz12-depleted cells [Boucher et al., 2004; data not shown]. In addition, p42/p44 MAPK phosphorylation levels were higher in serum-starved ShSuz12 cells stimulated with serum (data not shown). Thus, Suz12 depletion results in a decrease in IEC-6 cell density control, and increased independence to growth factors. This correlates with increased expression of positive G1 regulators of cell control. Furthermore, the absence of one PRC2 target, namely Cdkn2a [Maertens et al., 2009], reveals cell growth properties resulting from H3K27me inhibition.

### Suz12 Depletion in IEC-6 Cells Deregulates Differentiation-Specific Genes, Such as Neuronal Genes, and Inflammatory Genes

To determine the impact of Suz12 depletion on global IEC-6 cell gene expression patterns, we performed a gene expression microarray analysis. We selected genes significantly expressed (at least two strong signals (P) detected from triplicate and *P* < 0.05), by comparing gene expression in Suz12-depleted and control cells. Expression of 961 genes was increased at least two times in ShSuz12 IEC-6 cells. In contrast, 124 genes displayed twofold decreased expression. From the list of genes induced more than twofold in ShSuz12 IEC-6 cells, up to 667 genes were identified (Supplementary Table II).

Functional annotations from Gene Ontology were performed using independently the ToppGene and DAVID programs, and classification of genes according to the biological processes was analyzed. Categories considered strongly enriched (*P* < 0.05) were selected. Supplementary Figures 1 and 2 show the main classes of GO biological processes identified from the list of genes induced more than fivefold in ShSuz12 IEC-6 cells according to ToppGene Suite and DAVID database respectively (*P* < 0.05). The most significant classes of biological processes identified from ToppGene Suite and DAVID database were selected, and the list of genes associated with each class is shown in Supplementary Tables III and IV. Overall, the most significantly induced groups included biological processes related to cell adhesion, immune responses, cell proliferation, and development.

**Fig. 4:**
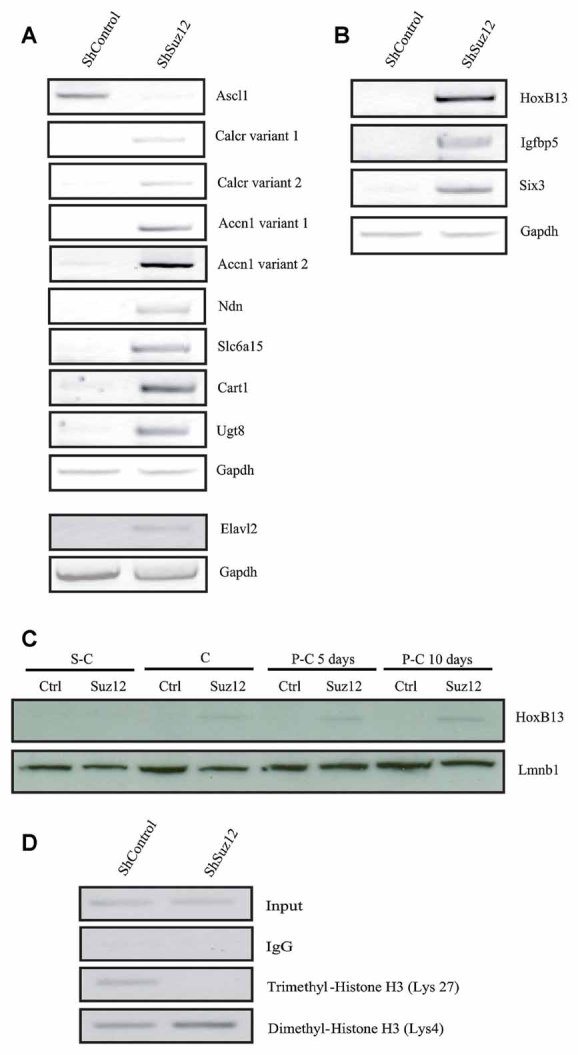
Suz12 depletion deregulates differentiation-specific genes. A,B: RNA was isolated from control or Suz12 depleted cells. Expression levels of various neuronal genes were verified by semi-quantitative RT-PCR, with Gapdh expression as a loading control. The amplified products were separated on 1.5% agarose gels. The results are representative of two independent experiments. C: Twenty microgram of nuclear protein extracts from sub-confluent (s-c), confluent (c), 5- or 10-day post-confluent (p-c) ShControl (Ctrl) and ShSuz12 IEC-6 cells were separated by SDS–PAGE and transferred to PVDF membranes for Western blot analysis of HoxB13 and Lmnb1 as a loading control. The results are representative of two independent experiments. D: Chromatin immunoprecipitation assays (ChIP) were performed with chromatin extracts from ShControl and ShSuz12 cells. Chromatin was immunoprecipitated with IgG, or with antibodies recognizing H3K27 trimethylation and H3K4 dimethylation marks. ChIP samples were verified by PCR analysis with oligonucleotides amplifying rat proximal promoter regions of upregulated gene necdin (Ndn). DNA levels were determined by PCR using 1% of input DNA prior to immunoprecipitation. The amplified products were separated on 2% agarose gels. Results are representative of two independent experiments.

From the microarray data, we selected various neuronal genes either upregulated (Ndn, Slc6a15, Cart1, Ugt8, Calcr, Accn1, Elavl2), or downregulated (Ascl1). Semi-quantitative RT-PCR analysis confirmed the increased pattern of expression of these genes, as opposed to Ascl1 (Fig. 4A). RT-PCR analysis also showed that Suz12 depletion increased the expression of a subset of Hox genes, including HoxB13 and Six3, and of the Igfbp5 gene (Fig. 4B). In murine embryonic stem cells, in contrast to the Hoxb13 and Elavl2 genes associated respectively with H3K27 or H3K4 methylation, the other genes display a bivalent H3K4/H3K27 methyl mark [Ku et al., 2008; Supplementary Table IV].

While these genes were transcriptionally activated in Suz12-depleted cells, we verified the pattern of expression of one selected gene, namely HoxB13, at the protein level. HoxB13 expression is associated with different tumorigenic processes in ovarian [Miao et al., 2007], breast [Jerevall et al., 2008], and prostate carcinomas [Jung et al., 2004]. Western blot analysis of nuclear proteins showed that HoxB3 was indeed expressed, and that protein levels increased in Suz12-depleted cells after reaching confluence (Fig. 4C).

We determined by chromatin immunoprecipitation assays, the status of the H3K27 methylation mark on the Ndn promoter, which displays a bivalent H3K4/H3K27 mark in embryonic stem cells [Ku et al., 2008], and whose expression is induced in Suz12-depleted cells. We also verified the H3K4 dimethylation modification associated with an active transcriptional state. Results show that while the Ncd proximal promoter H3K27 trimethylation mark was decreased in Suz12-depleted cells as opposed to control cells, basal H3K4 dimethylation levels were increased (Fig. 4D). Thus, Suz12 depletion de-represses non-specific differentiation pathways in IEC-6 cells, primarily by reducing the H3K27 methylation mark.

### Suz12 Depletion in IEC-6 Cells Modifies β-Catenin and STAT3 Activation

**Fig. 5:**
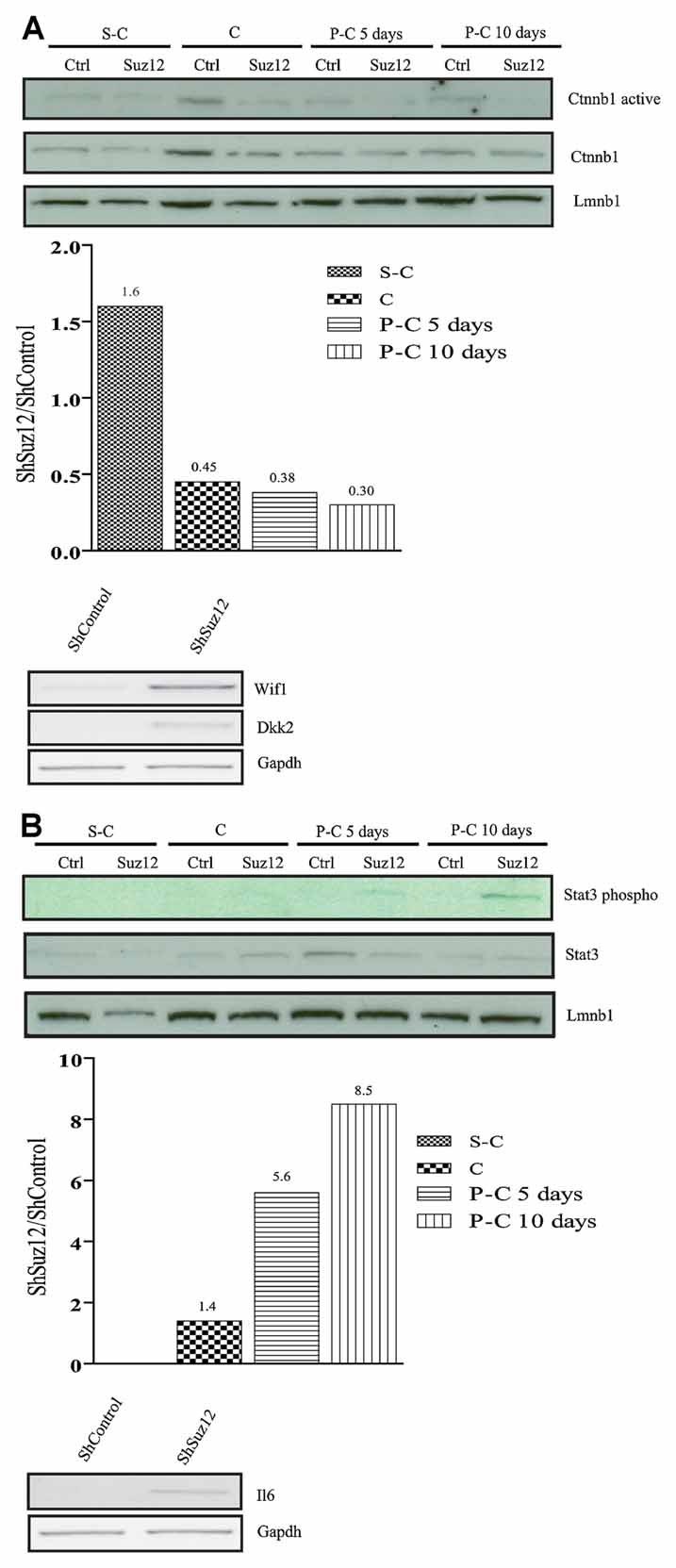
Distinct responses of β-catenin and Stat3 pathways in Suz12 depleted cells. Twenty microgram of nuclear protein extracts from sub-confluent (s-c), confluent (c), 5- or 10-day post-confluent (p-c) ShControl (Ctrl) and ShSuz12 IEC-6 cells were separated by SDS–PAGE and transferred to PVDF membranes for Western blot analysis of active β-catenin and β-catenin (A) or phospho-Stat3 and Stat3 (B) with Lmnb1 as a loading control. The histograms indicate the ratio of sub-confluent and confluent shSuz12 versus ShControl band intensities normalized to actin. Relative quantification of band intensity was performed with the Quantity One software. Results are representative of two independent experiments. In addition, total RNAs were isolated from control or Suz12 depleted cells. Expression levels of genes involved in Wnt pathway regulation (Wif1, Dkk2; A) or in STAT3 activation (Il6; B) were verified by semi-quantitative RT-PCR, with Gapdh expression as a loading control. The amplified products were separated on 1.5% agarose gels. Results are representative of two independent experiments.

We analyzed the microarray data with the MsigDB database to identify signaling pathways that could be affected in Suz12-depleted IEC-6 cells. Of 36 pathways significantly altered, three affected signaling pathways regulating cell proliferation and/or inflammation, namely Wnt signaling (12 out of 65 genes, 9.56E−3), IL-6 (5 out of 21 genes, 3.72E−2), and Jak/Stat signaling pathways (10 out of 153 genes, 4.09E−2), and MAPK signaling pathway (16 out of 257 genes, 9.56E−3). As Stat3 and β-catenin regulate cell proliferation, we thus verified by Western blot, the nuclear expression of activated phospho-β-catenin (Ctnnb1), with an antibody recognizing the non-phosphorylated protein, and phospho-Stat3 in sub-confluent, confluent, 5- and 10-day confluent control and Suz12-depleted cells. While activated Ctnnb1 levels were higher in sub-confluent Suz12-depleted cells as opposed to control cells, confluency led to decreased activated Ctnnb1 nuclear levels primarily in Suz12-depleted cells (Fig. 5A). This negative regulation correlated with overexpression of Wnt signaling pathway inhibitors, namely Wif1 and Dkk2, as shown by microarray analysis and semi-quantitative RT-PCR analysis (Fig. 5A). In contrast to decreased activated Ctnnb1 levels, while nuclear phosphorylated Stat3 levels were minimal in control cells, Stat3 phosphorylation increased in post-confluent Suz12-depleted cells (Fig. 5B). This positive regulation could be caused by the overexpression of Stat signaling pathway ligands. Indeed, microarray analysis and semi-quantitative RT-PCR analysis show that the Stat3 ligand Il6 was induced in Suz12-depleted cells. Thus, Suz12 depletion, by regulating the expression of β-catenin and Stat3 activity modulators, may modify IEC-6 cell dependence to proliferation pathways implicating β-catenin and Stat3.

### Suz12 Depletion in IEC-6 Cells Affects IL-1β-Dependent Regulation of Inflammatory Genes

**Fig. 6:**
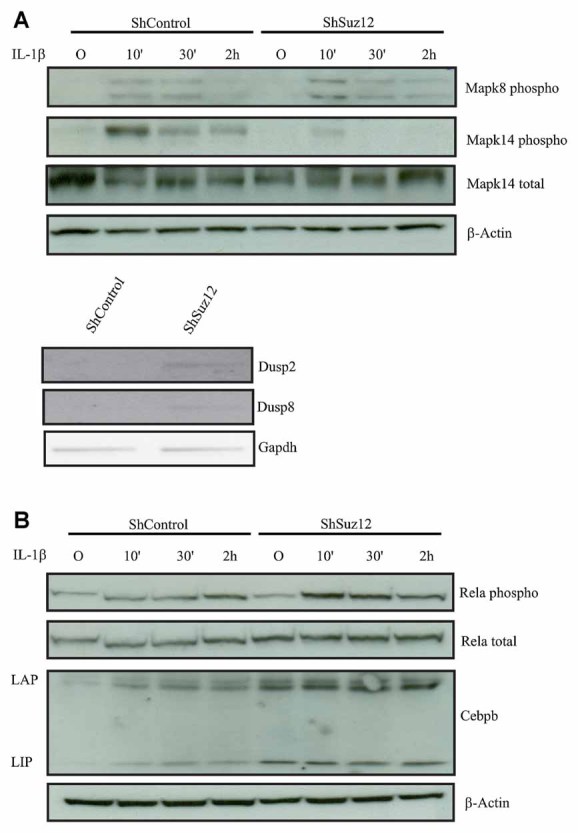
Distinct responses of MAPK, NF-κB p65 Rela, and Cebpb activation in response to IL-1β in Suz12 depleted cells. Control or Suz12 depleted IEC-6 cells were stimulated with 10 ng/ml of IL-1β for 10 min, 30 min, and 2 h. Whole cell protein extracts were separated by SDS–PAGE and transferred to PVDF membranes for Western blot analysis of phosphorylated JNK (Mapk8) and of total and phosphorylated p38 MAPK (Mapk14; A), and of total and phosphorylated-NF-κB p65 (Rela), and C/EBPβ (Cebp; B) with actin as a loading control. Results are representative of three independent experiments. In addition, total RNAs were isolated from control or Suz12 depleted cells. Expression levels of genes involved in MAPK pathway regulation (Dusp2, Dusp8; A) were verified by semi-quantitative RT-PCR, with Gapdh expression as a loading control. The amplified products were separated on 1.5% agarose gels. Results are representative of two independent experiments.

Inflammatory genes are one of the major classes of genes increased after Suz12 depletion, as determined by microarray analysis. We first assessed whether Suz12 depletion affected JNK (Mapk8) and p38 (Mapk14) MAPK phosphorylation in response to IL-1β. While both control and Suz12-depleted cells displayed the same level of JNK activation, p38 activation was consistently lower in Suz12-depleted cells (Fig. 6A). This correlated with increased expression of Dusp2 and Dusp8 dual specificity MAPK phosphatase, as assessed by microarray and semi-quantitative RT-PCR analysis. We then verified protein levels of inflammatory gene regulators, namely NF-κB p65 (Rela) and its phosphorylated form, and C/EBPβ. IL-1β stimulated Rela phosphorylation in both cell lines, with higher phosphorylation levels achieved in Suz12-depleted cells (Fig. 6B). Basal C/EBPβ protein levels were increased in Suz12-depleted cells (Fig. 6B). However, these increased levels were not induced by IL-1β, in contrast to control cells. Thus, Suz12 depletion may alter IEC-6 cell response to inflammatory stimuli.

**Fig. 7:**
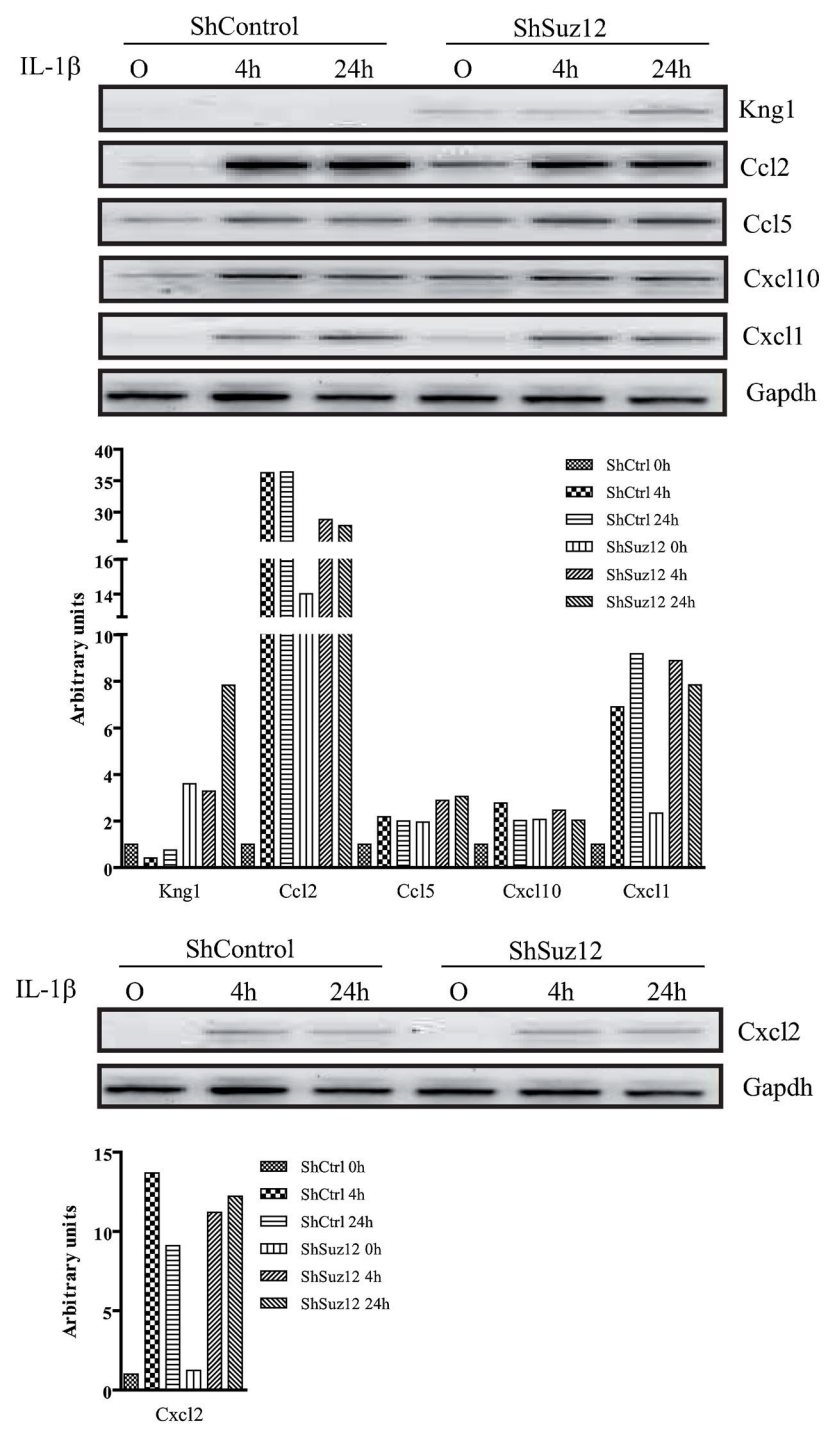
Suz12 depletion deregulates basal and IL-1β-induced inflammatory gene expression. Total RNAs were isolated from control or Suz12 depleted IEC-6 cells stimulated with 10 ng/ml of IL-1β for 4 or 24 h. Expression levels of various IL-1β-regulated inflammatory genes were verified by semi-quantitative RT-PCR, with Gapdh expression as a loading control. The amplified products were separated on 1.5% agarose gels. The histograms indicate the ratio of ShSuz12 versus ShControl band intensities normalized to Gapdh. Relative quantification of band intensity was performed with the Quantity One software. Results are representative of two independent experiments.

We then verified the expression of a panel of IL-1β-regulated inflammatory genes, including chemokines and acute phase protein genes, by semi-quantitative RT-PCR analysis, after 4 and 24 h of IL-1β induction. We observed various patterns of expression. Cxcl2 basal as well as IL-1β-induced levels were not affected by Suz12 depletion, in comparison to control cells (Fig. 7). In contrast, Suz12 depletion led to increased basal levels of Lcn2, Ccl20, and Lbp (not shown), and of Ccl2, Ccl5, Cxcl1, and Cxcl10 (Fig. 7), while IL-1β-induced levels were increased at similar levels. Whereas IL-1β treatment did not induce significantly Kng1 expression in control cells, Suz12 depletion resulted in increased basal and IL-1β-induced levels (Fig. 7). Induction of an inflammatory response with either TNFα or flagellin, a bacterial product activating Tlr5, gave similar results (data not shown). We then determined the basal pattern of expression of a panel of cytokines and chemokines with a protein array, in confluent control and Suz12-depleted cells. The results show that the basal levels of some cytokines and chemokines, including Cxcl1 and Ccl5, were increased (Supplementary Fig. 3). This correlated with Cxcl1 and Ccl5 RNA increases in Suz12-depleted cells (Fig. 7). In contrast, both Cxcl2 protein and RNA levels were not affected. However, in contrast to RNA levels, Il6 protein levels were not increased. Thus, Suz12 depletion leads to general increases of inflammatory gene RNA basal levels as well as specific cytokine and chemokine protein levels, and modifies the IL-1β response.

## DISCUSSION

Our results support a role for the H3K27methyl mark as a regulator of cell-density dependent proliferation. As found in other cell types, we found that PcG component expression is higher in intestinal crypt proliferative cells, as opposed to differentiated cells. Indeed, Suz12 as well as Ezh2 are mostly expressed in IEC crypt cells. Suz12 down-regulation led to decreases in Ezh2 expression and H3K27 methylation. Suz12 interaction with Ezh2 is important both for its enzymatic activity and protein stability [Jones and Wang, 2010].

The PRC2 polycomb complex regulates cell growth through the G1/S phase by repressing, among others, the cell cycle inhibitor Cdkn2a. PcG genes are overexpressed in many cancers, and are considered oncogenic in part, by their repressing effect, notably on Cdkn2a [Sauvageau and Sauvageau, 2010]. We show that the absence of Cdkn2a in Suz12-depleted IEC-6 cells reveals growth suppressing properties of the H3K27methyl mark. Indeed, Suz12-depleted cells grew to high cell densities. Growth was characterized by increased H3S10 phosphorylation, a histone mark correlating with cell growth, as well as higher Ccnd2 and Ccnd3 protein levels. We have identified two signaling pathways involved in cell growth which are differently regulated in the absence of Suz12. While Suz12-depleted cells showed decreased levels of activated β-catenin (Ctnnb1) as compared to control cells, Stat3 activity increased in a cell-density dependent manner. Decreased β-catenin signaling was associated with increased expression of Wnt signaling inhibitors, namely Wif1 and Dkk2, whose promoters contain the dual H3K4/H3K27 methylation mark in embryonic stem cells [Ku et al., 2008; see Supplementary Table V]. The Stat3 transcription factor plays a dual role as a regulator of cell proliferation and inflammation. Indeed, tumor cells are characterized by sustained Stat3 activation [Yu et al., 2009]. In Suz12-depleted cells, Stat3 activation may be sustained by autocrine signaling, since the expression of Il6, a known Stat3 inducer, is elevated. Of note, Il6 expression is independent of the H3K27methyl mark since the Il6 promoter region is not associated with H3K4 or H3K27 methylation, at least in murine embryonic stem cells [Ku et al., 2008; see Supplementary Table V]. Interestingly, mutations of a number of PcG genes in *Drosophila* eye discs lead to JAK-STAT pathway-dependent increased proliferation, in part through up-regulation of the JAK Hopscotch receptor ligand, named unpaired (Upd) [Classen et al., 2009; Wang and Huang, 2010]. However, no increase in Il6 protein secretion was observed. Thus, other chemokines or cytokines may be involved in maintaining Stat3 activation. For example, Cxcl1 and Cxcl5, whose expression is induced through Stat3 activity, may indirectly activate Stat3 through their interaction with the Cxcr2 receptor [Burger et al., 2005]. Thus, in addition to oncogenic roles, PcG proteins may function as tumor suppressors by inhibiting specific signaling pathways regulating cell growth, such as Stat3 signaling. Indeed, density-dependent cell growth of Suz12-depleted intestinal epithelial cells may become independent of β-catenin signaling, while requiring sustained Stat3 activation through autocrine signaling.

Our results support a role for the H3K27methyl mark as a regulator of intrinsic inflammatory responses. In fact, microarray data, as well as semi-quantitative RT-PCR analysis, show that many inflammatory genes were significantly induced. Basal inflammatory gene expression may be indirectly regulated in Suz12-depleted cells. Indeed, transcription factors regulating inflammatory gene expression, such as activated Stat3 and C/EBPβ, were induced. In addition, while some inflammatory genes, such as Cxcl1 and Cxcl10, contain a H3K27 mark in embryonic stem cells [Ku et al., 2008; see Supplementary Table V], others do not (Kng1, Ccl2, Ccl5). Increased basal levels may be achieved by transcriptional as well as post-transcriptional mechanisms. Suz12-deficient cells responded to IL-1β stimulation, with increased short-term Rela activation. However, while similar increased inflammatory gene mRNA levels were attained after IL-1β stimulation, induction levels remained higher in control cells, when compared to basal expression. Interestingly, Suz12-depleted cells show decreased Mapk14 (p38) phosphorylation in response to IL-1β, as opposed to Jnk (Mapk8). This correlates with increases in dual specificity phosphatases Dusp2 and Dusp 8. The p38 MAPK is involved in the inflammatory responses induced by many mediators, including IL-1β [Weber et al., 2010]. Interestingly, it has been shown that p38 phosphorylates Ezh2 in skeletal muscle cells, leading to increased PRC2-dependent repression of cell lineage markers, such as Pax7 [Palacios et al., 2010; Caretti et al., 2011]. Again, in contrast to the inhibition of Stat3 signaling, the PRC2 complex may insure the activation of the p38 MAPK pathway by repressing negative regulators of this pathway.

In conclusion, we have shown that the H3K27 mark may control cell proliferation as well as the inflammatory response by regulating specific signaling pathways, either negatively for the Stat3 pathway, or positively for the p38 MAPK pathway. Small modifications in PRC2 activity leading to variations in H3K27 methylation, in the context of genetic disturbances such as Cdkn2a inactivation, could result in activation or repression of parallel pathways of gene activity, leading to increased proliferation as well as intrinsic inflammatory gene stimulation.
