# Supplementary material for: The Histone H3K27 Methylation Mark Regulates Intestinal Epithelial Cell Density-Dependent Proliferation and the Inflammatory Response
Source: J Cell Biochem. 2012 Nov 28;114(5):1203–15. doi: 10.1002/jcb.24463 (PMC3617464; doi:10.1002/jcb.24463)
Supplement: Supplementary file 2 [file jcb0114-1203-SD2.doc]

**Supplementary Fig. 2.** Classification of genes induced more than 5 times in Suz12 depleted cells according to their GO biological process, using DAVID database (Database for Annotation, Visualization and Integrated Discovery).

**
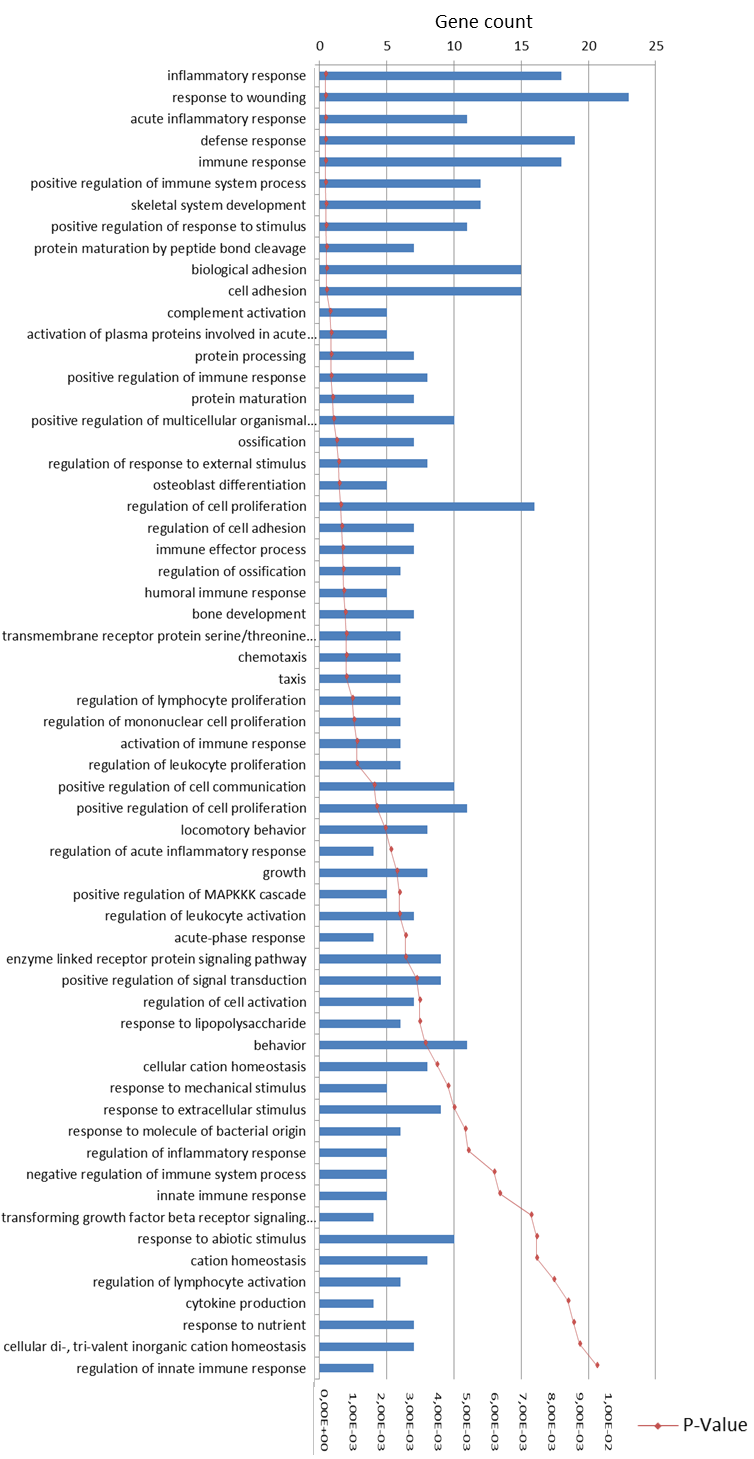
**
