# Supplementary material for: The Histone H3K27 Methylation Mark Regulates Intestinal Epithelial Cell Density-Dependent Proliferation and the Inflammatory Response
Source: J Cell Biochem. 2012 Nov 28;114(5):1203–15. doi: 10.1002/jcb.24463 (PMC3617464; doi:10.1002/jcb.24463)
Supplement: Supplementary file 4 [file jcb0114-1203-SD4.doc]

**FIGURE LEGENDS FOR SUPPLEMENTARY DATA**

**Supplementary Figure 1. Classification of genes induced more than five-fold in Suz12-depleted IEC-6 cells according to their GO biological process, using the ToppGene Suite.**

**Supplementary Figure 2. Classification of genes induced more than five-fold in Suz12-depleted IEC-6 cells according to their GO biological process, using the DAVID database.**

**Supplementary Figure 3. Suz12 depletion deregulates basal cytokine and chemokine protein expression. A.** Supernatants from 6 independent confluent control and Suz12 depleted IEC-6 cell cultures were pooled. The sample and antibody mixture was incubated with the Rat Cytokine Array Panel A membrane (Proteome Profiler Array, R&D Systems, Minneapolis, MN). The immune complex was revealed by Streptavidin-HRP and chemiluminescent detection reagents. Data shown are from a 5 minute exposure to X-ray film. The array membranes correspond to control (ShControl) and Suz12 depleted cells (ShSuz12). Cytokines and chemokines that show increased expression are indicated by numbers, namely 1 (Cxcl1), 2 (Cxcl2), 3 (Cx3cl1), 4 (Cxcl5). 5 (Ccl20), 6 (Ccl5) and 7 (Il6). **B.** Fluorescence intensity was measured with a Microarray scanner. The histogram represents the fluorescence intensity for Cxcl1, Cxcl2, Cx3cl1, Cxcl5, Ccl20, Ccl5 and Il6.

**TABLE LEGENDS FOR SUPPLEMENTARY DATA**

**Supplementary Table 1. A. Oligonucleotides used for semi-quantitative RT-PCR. B. Oligonucleotides used for chromatin immunoprecipitation.**

**Supplementary Table 2. List of genes induced more than two-fold in Suz12-depleted IEC-6 cells.**

**Supplementary Table 3. List of genes induced more than five-fold in Suz12-depleted IEC-6 cells, according to their GO biological process, using the ToppGene Suite.**

**Supplementary Table 4. List of genes induced more than five-fold in Suz12-depleted IEC-6 cells, according to their GO biological process, using the DAVID database.**

**Supplementary Table 5. Status of H3K4 and H3K27 methylation of genes analysed in this study, in murine ES cells (Ku *et al*., 2008).**
