# Supplementary material for: The Histone H3K27 Methylation Mark Regulates Intestinal Epithelial Cell Density-Dependent Proliferation and the Inflammatory Response
Source: J Cell Biochem. 2012 Nov 28;114(5):1203–15. doi: 10.1002/jcb.24463 (PMC3617464; doi:10.1002/jcb.24463)
Supplement: Supplementary file 5 [file jcb0114-1203-SD5.doc]

Supplementary Table 1A. Oligonucleotides used for semi-quantitative RT-PCR

|  |  |  |  |  |
| --- | --- | --- | --- | --- |
| Gene | UP | Down | Accession |  |
|  |  |  |  |  |
| Ccdn1 | 5’-TGATGGCATTACCTGGACAA-3’ | 5’-GCAAGAATGTGCCAGACTCA-3’ | NM_171992 |  |
| Ccdn2 | 5’-TTACCTGGACCGTTTCTTGG-3’ | 5’-GGTAGCACACAGAGCGATGA-3’ | NM_022267 |  |
| Cdkn1c | 5’-TCTGACCTCAGACCCGATTC-3’ | 5’-CCTTCAGCTCCTGATTCTCG-3’ | NM_182735 |  |
| Cdkn1a | 5’-TCAGTGGACCAGAAGGGAAC-3’ | 5’-GGGAGGGGACCAAAATACAT-3’ | NM_080782 |  |
| Cdkn1b | 5’-TTGCGCAATTAGGTTTTTCC-3’ | 5’-CAACTCCCTGTGGCGATTAT-3’ | NM_031762 |  |
| Ascl1 | 5'-GGCTCAACTTCAGTGGCTTC-3' | 5'-ACACAGGATCTCCTGCCATC-3' | NM_022384 |  |
| Calcr variant 1 | 5'-TTTCCAGGGATTCTTTGTCG-3' | 5'-GCCTGACTCCCAAAGCAATA-3' | NM_053816 |  |
| Calcr variant 2 | 5'-CCCCAGAATGAGATCTTCCA-3' | 5'-TTGTACCAGAGCTGCCTGAA-3' | NM_001034015 |  |
| Accn1 variant 1 | 5'-CGGTCTACTGGCAGAAAAGG-3' | 5'-AGTGGTTTGGCATTGTGTCA-3' | NM_012892 |  |
| Accn1 variant 2 | 5'-TACAAAGCACCTGCATGCTC-3' | 5'-CCGCCCTAAACAAAACAAAA-3' | NM_001034014 |  |
| Ndn | 5'-TGGTACGTGTTGGTCAAGGA-3' | 5'-GGACGAACTCCTCGGTGATA-3' | NM_001008558 |  |
| Slc6a15 | 5'-GTCCCAGTCGTTTTCGTCAT-3' | 5'-TAAAGATGGCGGCTATGGAC-3' | NM_172321 |  |
| CART 1 | 5'-AGCGGTGGTTCTGTGGTTAC-3' | 5'-CAGTCCTTTCAGGGAATCCA-3' | NM_012921 |  |
| Ugt8 | 5'-TACGGCTCAAACAGCACTTG-3' | 5'-GTTCAACAGGGAGGTCCTGA-3' | NM_019276 |  |
| HoxB13 | 5'-AGGTGAACAGAACCCACCAG-3' | 5'-TGAAGGGGACTGTGATTTCC-3' | NM_001107041 |  |
| Igfbp5 | 5'-GCTCTTTCGTGCATTGTGAA-3' | 5'-ATCTCAGGTGCAGGGATGAC-3' | NM_012817 |  |
| Six3 | 5'-TACGGCTCAAACAGCACTTG-3' | 5'-GTTCAACAGGGAGGTCCTGA-3' | NM_023990 |  |
| Elavl2 | 5'-CAGTGCATCAGATTCGTTGG-3' | 5'-TCAGCATGAAAACTCGCTTG-3' | NM_173309 |  |
| Wif1 | 5’-GGACTTGAGGGAGAGCAGTG-3’ | 5’-CCGTTTCAGATGGTGGAGTT-3’ | NM_053738 |  |
| Dkk2 | 5’-CAGGGGGAAGTCTGTACCAA-3’ | 5’-GTGCACAATGGAAACACTGG-3’ | NM_001106472 |  |
| Il6 | 5’-CCGGAGAGGAGACTTCACAG-3’ | 5’-GGTTTGCCGAGTAGACCTCA-3’ | NM_012585 |  |
| Dusp2 | 5’-CTCCCCCAACTTCAGTTTCA-3’ | 5’-GCTTGTTTTCTGAGCCCTTG-3’ | NM_001012089 |  |
| Dusp8 | 5’-GCCAGATGGAGTTCGAAGAG-3’ | 5’-GGATTGAAGGTGGCCTCATA-3’ | NM_001108510 |  |
| Kng1 | 5'-TTCTGAAACACGCTGTGGAG-3' | 5'-CATTGCAGTTGAGGCTTTGA-3' | NM_012741 |  |
| Ccl2 | 5'-CCAGAAACCAGCCAACTCTC-3' | 5'-AGGCATCACATTCCAAATCA-3' | NM_031530 |  |
| Slpi | 5'-GTGGAAGGAGGCAAAAATGA-3' | 5'-TTGGGAGGGTTAAGCATCAG-3' | NM_053372 |  |
| Ccl5 | 5’-GTGCCCACGTGAAGGAGTAT-3’ | 5’-ATCCCCAGCTGGTTAGGACT-3’ | NM_031116 |  |
| Cxcl10 | 5’-TGTCCGCATGTTGAGATCAT-3’ | 5’-ATTTGCCATCTCACCTGGAC-3’ | NM_139089 |  |
| Cxcl1 | 5’-CCCCATGGTTCAGAAGATTG-3’ | 5’-AGGCATTGTGCCCTACAAAC-3’ | NM_030845 |  |
| Cxcl2 | 5’-CCTCCTGTGCTCAAGACTCC-3’ | 5’-GAGCTGGCCAATGCATATCT-3’ | NM_053647 |  |
| GAPDH | 5'-CCAAAGTTGTCATGGATGAC-3' | 5'-GTGAAGGTCGGTGTGAACGG-3' | NM_017008 |  |

Supplementary Table 1B. Oligonucleotides used for chromatin immunoprecipitation

|  |  |  |  |  |
| --- | --- | --- | --- | --- |
| Gene | Region | UP | Down | Accession |
|  |  |  |  |  |
|  |  |  |  |  |
| Ndn | -16 to -230 | 5'-TCCCCACTATCACCCATCTC -3' | 5'-GTGCGCTTTACTGAGCACTG -3' | NM_001008558 |
