# Supplementary material for: The Histone H3K27 Methylation Mark Regulates Intestinal Epithelial Cell Density-Dependent Proliferation and the Inflammatory Response
Source: J Cell Biochem. 2012 Nov 28;114(5):1203–15. doi: 10.1002/jcb.24463 (PMC3617464; doi:10.1002/jcb.24463)
Supplement: Supplementary file 6 [file jcb0114-1203-SD6.doc]

**Supplementary Table 2.** List of genes induced more than 2 times in Suz12 depleted cells.

| **Probeset** | **Rep. Public ID** | **Gene Title** | **Gene Symbol** | **Fold change** | **P-value** |
| --- | --- | --- | --- | --- | --- |
| 1380828_at | AI145413 | gamma-aminobutyric acid (GABA) A receptor, alpha 1 | Gabra1 | 111,67 | 8,33E-07 |
| 1371947_at | BG671865 | necdin homolog (mouse) | Ndn | 64,53 | 5,12E-07 |
| 1368806_at | AA799627 | selenoprotein P, plasma, 1 | Sepp1 | 48,72 | 5,90E-07 |
| 1373333_at | AI102732 | similar to Microsomal signal peptidase 23 kDa subunit (SPase 22 kDa subunit) (SPC22/23) | MGC109340 | 40,84 | 1,55E-08 |
| 1387868_at | BF289368 | lipopolysaccharide binding protein | Lbp | 38,44 | 1,22E-07 |
| 1373386_at | AI179953 | gap junction protein, beta 2 | Gjb2 | 35,18 | 2,49E-07 |
| 1373458_at | BI289546 | brain expressed gene 4 | Bex4 | 34,63 | 1,40E-06 |
| 1367794_at | NM_012488 | alpha-2-macroglobulin | A2m | 33,50 | 1,02E-06 |
| 1367581_a_at | AB001382 | secreted phosphoprotein 1 | Spp1 | 31,57 | 7,24E-08 |
| 1387968_at | L22022 | solute carrier family 6 (neutral amino acid transporter), member 15 | Slc6a15 | 28,02 | 3,04E-06 |
| 1372818_at | BI284441 | collectin sub-family member 12 | Colec12 | 27,12 | 3,90E-07 |
| 1372440_at | BI275818 | serine (or cysteine) peptidase inhibitor, clade E, member 2 | Serpine2 | 26,40 | 2,87E-05 |
| 1387488_a_at | L13041 | calcitonin receptor | Calcr | 25,32 | 1,06E-05 |
| 1387131_at | AF193015 | serine (or cysteine) peptidase inhibitor, clade I, member 1 | Serpini1 | 23,64 | 1,20E-06 |
| 1369203_at | NM_053738 | Wnt inhibitory factor 1 | Wif1 | 22,29 | 1,30E-06 |
| 1375984_at | BE103689 | zinc finger homeobox 4 | Zfhx4 | 20,73 | 6,74E-07 |
| 1388711_at | BF282650 | interleukin 13 receptor, alpha 1 | Il13ra1 | 20,43 | 3,79E-06 |
| 1387029_at | NM_130409 | complement factor H | Cfh | 19,95 | 5,38E-05 |
| 1370125_at | NM_019189 | hyaluronan and proteoglycan link protein 1 | Hapln1 | 19,16 | 2,99E-06 |
| 1367571_a_at | NM_031511 | insulin-like growth factor 2 | Igf2 | 18,80 | 2,58E-05 |
| 1388802_at | AI579422 | brain expressed gene 1 | Bex1 | 18,24 | 2,45E-06 |
| 1367973_at | NM_031530 | chemokine (C-C motif) ligand 2 | Ccl2 | 17,87 | 3,21E-06 |
| 1372390_at | AI710604 | Antisense paternally expressed gene 3 | Apeg3 | 17,52 | 1,13E-04 |
| 1368533_at | NM_133304 | hephaestin | Heph | 17,39 | 1,62E-05 |
| 1368541_at | NM_053719 | embigin homolog (mouse) | Emb | 17,26 | 1,41E-06 |
| 1389735_at | BE107296 | Ribosomal protein S6 kinase polypeptide 6 | Rps6ka6 | 16,49 | 2,11E-06 |
| 1386160_at | AI639401 | Trichohyalin | Tchh | 16,43 | 1,51E-06 |
| 1376198_at | BI303342 | adipocyte-specific adhesion molecule | Asam | 16,25 | 1,81E-07 |
| 1388142_at | AA850991 | versican | Vcan | 16,01 | 1,27E-06 |
| 1379626_at | AA946353 | SATB homeobox 1 | Satb1 | 15,19 | 6,83E-06 |
| 1368256_at | NM_053779 | serine (or cysteine) peptidase inhibitor, clade I, member 1 | Serpini1 | 14,98 | 1,23E-07 |
| 1367733_at | NM_019291 | carbonic anhydrase II | Car2 | 14,58 | 1,46E-06 |
| 1384541_at | BM391441 | hyaluronan and proteoglycan link protein 1 | Hapln1 | 14,44 | 2,63E-06 |
| 1372374_at | BM383006 | carbonic anhydrase 1 | Car1 | 14,22 | 1,05E-05 |
| 1392647_at | BF282318 | Similar to Serum amyloid A-3 protein precursor | LOC691143 | 14,21 | 1,44E-06 |
| 1383575_at | BG376561 | Catenin (cadherin-associated protein), delta 2 (neural plakophilin-related arm-repeat protein) | Ctnnd2 | 14,10 | 6,21E-07 |
| 1380387_at | BE105492 | Forkhead box P2 | Foxp2 | 13,78 | 1,83E-09 |
| 1371696_at | AI412938 | G protein-coupled receptor 56 | Gpr56 | 12,91 | 8,82E-06 |
| 1368028_at | NM_012633 | peripherin | Prph | 12,86 | 1,86E-06 |
| 1392965_a_at | AI028877 | SPARC related modular calcium binding 2 | Smoc2 | 12,58 | 6,74E-06 |
| 1383783_at | AW521192 | protocadherin 9 | Pcdh9 | 12,35 | 1,66E-06 |
| 1367700_at | NM_080698 | fibromodulin | Fmod | 12,07 | 4,57E-08 |
| 1373401_at | AI176034 | Tenascin C | Tnc | 11,86 | 1,24E-05 |
| 1370960_at | BE104060 | insulin-like growth factor binding protein 5 | Igfbp5 | 11,74 | 5,67E-05 |
| 1370913_at | AI409634 | radical S-adenosyl methionine domain containing 2 | Rsad2 | 11,60 | 1,08E-04 |
| 1373911_at | BM389026 | periostin, osteoblast specific factor | Postn | 11,15 | 8,78E-07 |
| 1390942_at | AI043817 | Pellino 2 | Peli2 | 11,07 | 1,45E-05 |
| 1387050_s_at | NM_012696 | kininogen 1 /// kininogen 1-like 1 /// kininogen 2 | Kng1 /// Kng1l1 /// Kng2 | 11,07 | 2,79E-05 |
| 1387843_at | NM_012561 | follistatin | Fst | 11,03 | 2,10E-05 |
| 1375582_at | AW531902 | zinc finger homeobox 4 | Zfhx4 | 10,69 | 1,46E-05 |
| 1369814_at | AF053312 | chemokine (C-C motif) ligand 20 | Ccl20 | 10,59 | 1,14E-05 |
| 1383768_at | BF563441 | ELAV (embryonic lethal, abnormal vision, Drosophila)-like 2 (Hu antigen B) | Elavl2 | 10,39 | 7,01E-07 |
| 1391714_at | BI290063 | pleiomorphic adenoma gene 1 | Plag1 | 10,15 | 1,28E-05 |
| 1370428_x_at | AJ249701 | RT1 class Ia, locus A2 /// RT1 class I, locus A3 /// RT1 class Ib, locus EC2 | RT1-A2 /// RT1-A3 /// RT1-EC2 | 10,14 | 5,32E-07 |
| 1367998_at | NM_053372 | secretory leukocyte peptidase inhibitor | Slpi | 10,09 | 1,11E-06 |
| 1388879_at | BG669292 | similar to ABI gene family, member 3 (NESH) binding protein | RGD1562717 | 9,77 | 1,40E-06 |
| 1376654_at | AW521378 | similar to RIKEN cDNA B130016O10 gene | RGD1308448 | 9,74 | 6,55E-06 |
| 1372584_at | BG672517 | cannabinoid receptor interacting protein 1 | Cnrip1 | 9,27 | 5,35E-07 |
| 1386922_at | AI408948 | carbonic anhydrase II | Car2 | 8,94 | 5,08E-05 |
| 1387018_at | NM_053770 | sorbin and SH3 domain containing 2 | Sorbs2 | 8,87 | 2,97E-06 |
| 1382467_at | C07140 | brain expressed gene 1 /// brain expressed X-linked 2 | Bex1 /// Bex2 | 8,85 | 1,72E-07 |
| 1393202_a_at | BF414160 | Insulin-like growth factor 2 mRNA binding protein 3 | Igf2bp3 | 8,67 | 4,07E-07 |
| 1377086_at | AI233530 | C1q and tumor necrosis factor related protein 3 | C1qtnf3 | 8,66 | 3,65E-05 |
| 1384525_at | AI549335 | dedicator of cytokinesis 11 | Dock11 | 8,66 | 1,37E-05 |
| 1367765_at | NM_022534 | transcobalamin 2 | Tcn2 | 8,58 | 4,45E-07 |
| 1368474_at | NM_012889 | vascular cell adhesion molecule 1 | Vcam1 | 8,56 | 1,79E-04 |
| 1368332_at | NM_133624 | guanylate binding protein 2 | Gbp2 | 8,44 | 6,45E-08 |
| 1390119_at | BF396602 | secreted frizzled-related protein 2 | Sfrp2 | 8,43 | 4,87E-05 |
| 1368536_at | NM_057104 | ectonucleotide pyrophosphatase/phosphodiesterase 2 | Enpp2 | 8,39 | 1,27E-06 |
| 1369649_at | AF400662 | calcium channel, voltage-dependent, alpha2/delta subunit 1 | Cacna2d1 | 8,36 | 2,38E-05 |
| 1370248_at | AA851939 | FXYD domain-containing ion transport regulator 6 | Fxyd6 | 8,33 | 2,59E-08 |
| 1387134_at | NM_053687 | schlafen 3 | Slfn3 | 8,33 | 1,43E-05 |
| 1370728_at | AY044251 | interleukin 13 receptor, alpha 1 | Il13ra1 | 8,30 | 8,30E-06 |
| 1370892_at | BI285347 | complement component 4, gene 2 /// complement component 4B (Chido blood group) | C4-2 /// C4b | 8,21 | 1,24E-05 |
| 1394699_at | BG671896 | Similar to transcription factor ONECUT2 | RGD1564677 | 8,11 | 8,70E-06 |
| 1371700_at | AI177059 | microfibrillar-associated protein 4 | Mfap4 | 8,09 | 7,77E-08 |
| 1377729_at | BG668988 | Elongation of very long chain fatty acids (FEN1/Elo2, SUR4/Elo3, yeast)-like 4 | Elovl4 | 8,06 | 1,31E-05 |
| 1383736_at | AI145457 | ELAV (embryonic lethal, abnormal vision, Drosophila)-like 2 (Hu antigen B) | Elavl2 | 7,98 | 7,78E-05 |
| 1368576_at | NM_012921 | ALX homeobox 1 | Alx1 | 7,76 | 1,96E-05 |
| 1387505_at | NM_013145 | guanine nucleotide binding protein (G protein), alpha inhibiting 1 | Gnai1 | 7,76 | 7,08E-06 |
| 1378674_at | BI293056 | engrailed homeobox 2 | En2 | 7,72 | 7,01E-05 |
| 1376788_at | AA818353 | death associated protein kinase 1 | Dapk1 | 7,70 | 9,24E-05 |
| 1380726_at | BI290633 | Asporin | Aspn | 7,47 | 2,57E-04 |
| 1387969_at | U22520 | chemokine (C-X-C motif) ligand 10 | Cxcl10 | 7,42 | 9,79E-06 |
| 1393868_at | AW535602 | protocadherin 10 | Pcdh10 | 7,40 | 1,22E-04 |
| 1387165_at | NM_019318 | v-maf musculoaponeurotic fibrosarcoma oncogene homolog (avian) | Maf | 7,18 | 7,08E-05 |
| 1388054_a_at | AF072892 | versican | Vcan | 7,17 | 3,38E-04 |
| 1374320_at | AI717113 | coagulation factor V (proaccelerin, labile factor) | F5 | 7,13 | 3,09E-05 |
| 1372455_at | AI410264 | tetraspanin 12 | Tspan12 | 7,10 | 3,14E-05 |
| 1368470_at | NM_012960 | gamma-glutamyl hydrolase (conjugase, folylpolygammaglutamyl hydrolase) | Ggh | 7,09 | 2,78E-06 |
| 1371913_at | BG379319 | transforming growth factor, beta induced | Tgfbi | 7,08 | 1,20E-06 |
| 1377828_at | BG672090 | Anoctamin 3 | Ano3 | 7,08 | 1,11E-04 |
| 1371209_at | AJ243338 | RT1 class I, locus CE5 | RT1-CE5 | 7,06 | 5,33E-06 |
| 1387487_a_at | NM_053816 | calcitonin receptor | Calcr | 7,05 | 1,22E-04 |
| 1368655_at | NM_020074 | serglycin | Srgn | 7,04 | 5,98E-06 |
| 1368785_a_at | NM_019334 | paired-like homeodomain 2 | Pitx2 | 6,99 | 2,09E-05 |
| 1368395_at | NM_012774 | glypican 3 | Gpc3 | 6,90 | 9,69E-07 |
| 1377659_at | BI302005 | myeloid leukemia factor 1 | Mlf1 | 6,89 | 2,21E-06 |
| 1387893_at | D88250 | complement component 1, s subcomponent | C1s | 6,86 | 2,89E-06 |
| 1392751_at | AI029410 | fibronectin type III domain containing 3C1 | Fndc3c1 | 6,78 | 8,47E-05 |
| 1377867_at | BM390001 | similar to Glutaminyl-peptide cyclotransferase precursor (QC) | RGD1562284 | 6,71 | 4,65E-05 |
| 1387715_at | NM_133537 | extracellular proteinase inhibitor | Expi | 6,63 | 1,62E-05 |
| 1370491_a_at | M38759 | histidine decarboxylase | Hdc | 6,53 | 1,32E-05 |
| 1382220_at | AI180454 | insulin-like growth factor 2 mRNA binding protein 2 | Igf2bp2 | 6,41 | 1,66E-05 |
| 1370823_at | AF387513 | BMP and activin membrane-bound inhibitor, homolog (Xenopus laevis) | Bambi | 6,35 | 1,40E-05 |
| 1371237_a_at | AF411318 | metallothionein 1a | Mt1a | 6,31 | 9,12E-08 |
| 1373886_at | BF284692 | receptor accessory protein 1 | Reep1 | 6,27 | 3,36E-05 |
| 1377639_at | BI275896 | adipocyte-specific adhesion molecule | Asam | 6,26 | 7,83E-05 |
| 1388271_at | BM383531 | metallothionein 2A | Mt2A | 6,25 | 6,08E-07 |
| 1373891_at | AI101009 | zinc finger, CCHC domain containing 12 | Zcchc12 | 6,22 | 1,23E-04 |
| 1372637_at | AI169241 | similar to trophinin isoform 1 | LOC683751 | 6,22 | 3,10E-06 |
| 1387899_at | U52102 | collapsin response mediator protein 1 | Crmp1 | 6,16 | 6,64E-05 |
| 1369665_a_at | AJ222813 | interleukin 18 | Il18 | 6,13 | 2,36E-04 |
| 1372153_at | BM385502 | keratin 15 | Krt15 | 6,01 | 2,80E-05 |
| 1388071_x_at | M24024 | RT1 class Ib, locus EC2 | RT1-EC2 | 5,95 | 8,67E-07 |
| 1372013_at | BG380285 | interferon induced transmembrane protein 1 | Ifitm1 | 5,93 | 1,22E-05 |
| 1383413_at | AW531481 | hedgehog acyltransferase-like | Hhatl | 5,92 | 1,98E-05 |
| 1388255_x_at | AJ243338 | RT1 class I, locus CE5 | RT1-CE5 | 5,90 | 1,21E-06 |
| 1370301_at | U65656 | matrix metallopeptidase 2 | Mmp2 | 5,89 | 3,05E-05 |
| 1387039_at | NM_030828 | glypican 1 | Gpc1 | 5,83 | 2,46E-08 |
| 1387349_at | NM_013028 | short stature homeobox 2 | Shox2 | 5,82 | 3,11E-05 |
| 1370048_at | NM_053936 | lysophosphatidic acid receptor 1 | Lpar1 | 5,76 | 7,06E-06 |
| 1378997_at | BM391684 | Eph receptor B6 | Ephb6 | 5,75 | 2,61E-05 |
| 1378002_at | BF401583 | heat shock protein 4 like | Hspa4l | 5,74 | 1,66E-05 |
| 1370043_at | NM_031753 | activated leukocyte cell adhesion molecule | Alcam | 5,63 | 5,60E-06 |
| 1383606_at | BI302544 | tandem C2 domains, nuclear | Tc2n | 5,61 | 1,16E-04 |
| 1389034_at | BI295179 | ubiquitin specific peptidase 18 | Usp18 | 5,58 | 2,12E-05 |
| 1367749_at | NM_031050 | lumican | Lum | 5,50 | 3,41E-04 |
| 1383488_at | AA817785 | similar to Forkhead box protein F1 (Forkhead-related protein FKHL5) (Forkhead-related transcription factor 1) (FREAC-1) (Hepatocyte nuclear factor 3 forkhead homolog 8) (HFH-8) | LOC687536 | 5,46 | 1,93E-04 |
| 1398270_at | AA944827 | bone morphogenetic protein 2 | Bmp2 | 5,39 | 9,01E-06 |
| 1389470_at | AI639117 | complement factor B | Cfb | 5,39 | 1,46E-04 |
| 1368348_at | NM_013034 | solute carrier family 6 (neurotransmitter transporter, serotonin), member 4 | Slc6a4 | 5,38 | 1,68E-05 |
| 1392183_at | AI547389 | Homeobox C9 | Hoxc9 | 5,36 | 1,47E-08 |
| 1369735_at | NM_057100 | growth arrest specific 6 | Gas6 | 5,36 | 4,97E-06 |
| 1370256_at | AA944349 | frizzled homolog 1 (Drosophila) | Fzd1 | 5,34 | 7,35E-05 |
| 1380852_at | AI112346 | RALY RNA binding protein-like | Ralyl | 5,33 | 1,08E-04 |
| 1368128_at | NM_031598 | phospholipase A2, group IIA (platelets, synovial fluid) | Pla2g2a | 5,29 | 5,90E-05 |
| 1374474_at | BE099085 | copine VIII | Cpne8 | 5,28 | 1,01E-04 |
| 1391345_at | BI293047 | BMP-binding endothelial regulator | Bmper | 5,28 | 5,66E-07 |
| 1387982_at | AF057025 | toll-like receptor 4 | Tlr4 | 5,25 | 7,20E-05 |
| 1376106_at | AI010157 | transmembrane protein 178 | Tmem178 | 5,24 | 1,44E-04 |
| 1368420_at | NM_012532 | ceruloplasmin | Cp | 5,22 | 2,34E-06 |
| 1385702_at | AA901151 | interferon activated gene 204 | Ifi204 | 5,16 | 2,08E-05 |
| 1373177_x_at | AI409900 | rCG56785-like /// hypothetical LOC363363 /// hypothetical gene supported by BC082068 /// hypothetical protein LOC680682 /// hypothetical protein LOC685369 /// similar to Discs large homolog 5 (Placenta and prostate DLG) (Discs large protein P-dlg) /// hypothetical protein LOC691793 | LOC100360189 /// LOC363363 /// LOC501476 /// LOC680682 /// LOC685369 /// LOC689362 /// LOC691793 | 5,14 | 3,04E-05 |
| 1384824_at | BF408099 | protocadherin 18 | Pcdh18 | 5,09 | 9,99E-05 |
| 1374016_at | AI502597 | lysophosphatidic acid receptor 1 | Lpar1 | 5,09 | 8,26E-05 |
| 1368131_at | NM_031808 | calpain 6 | Capn6 | 5,08 | 2,51E-05 |
| 1387854_at | BI282748 | collagen, type I, alpha 2 | Col1a2 | 5,05 | 7,13E-07 |
| 1370959_at | BI275716 | collagen, type III, alpha 1 | Col3a1 | 5,03 | 5,86E-06 |
| 1369983_at | NM_031116 | chemokine (C-C motif) ligand 5 | Ccl5 | 5,01 | 8,72E-07 |
| 1373674_at | BI283094 | microfibrillar associated protein 5 | Mfap5 | 4,98 | 7,97E-06 |
| 1368642_at | NM_031333 | cadherin 2 | Cdh2 | 4,98 | 1,96E-05 |
| 1388569_at | AI179984 | serine (or cysteine) peptidase inhibitor, clade F, member 1 | Serpinf1 | 4,98 | 1,42E-06 |
| 1385397_at | AA859085 | STEAP family member 4 | Steap4 | 4,97 | 1,82E-05 |
| 1387196_at | NM_022249 | KH domain containing, RNA binding, signal transduction associated 3 | Khdrbs3 | 4,97 | 1,56E-06 |
| 1371702_at | AI234044 | tetraspanin 7 | Tspan7 | 4,95 | 5,58E-05 |
| 1372299_at | AI013919 | cyclin-dependent kinase inhibitor 1C | Cdkn1c | 4,94 | 1,05E-04 |
| 1394668_at | BF409960 | cell adhesion molecule 3 | Cadm3 | 4,93 | 1,79E-04 |
| 1368542_at | NM_053583 | zinc finger protein 423 | Zfp423 | 4,93 | 3,38E-04 |
| 1370139_a_at | AB051214 | transient receptor potential cation channel, subfamily C, member 6 | Trpc6 | 4,92 | 5,03E-05 |
| 1387839_at | NM_012646 | RT1 class Ib, locus N1 /// RT1 class Ib, locus N2 | RT1-N1 /// RT1-N2 | 4,91 | 8,80E-06 |
| 1378629_at | AI227638 | SATB homeobox 1 | Satb1 | 4,90 | 1,76E-04 |
| 1374635_at | AW527151 | Hypothetical protein LOC689663 | LOC689663 | 4,90 | 4,97E-06 |
| 1385486_at | AW534737 | basonuclin 2 | Bnc2 | 4,90 | 1,30E-05 |
| 1385659_at | AA956596 | similar to adenomatosis polyposis coli down-regulated 1 | LOC682861 | 4,90 | 4,35E-05 |
| 1370047_at | NM_053535 | ectonucleotide pyrophosphatase/phosphodiesterase 1 | Enpp1 | 4,88 | 1,92E-07 |
| 1383112_at | BF550315 | trichorhinophalangeal syndrome I homolog (human) | Trps1 | 4,88 | 2,35E-05 |
| 1370964_at | BF283456 | argininosuccinate synthetase 1 | Ass1 | 4,87 | 1,55E-05 |
| 1368200_at | NM_134455 | chemokine (C-X3-C motif) ligand 1 | Cx3cl1 | 4,82 | 8,77E-04 |
| 1388300_at | AA892234 | microsomal glutathione S-transferase 3 | Mgst3 | 4,79 | 5,24E-06 |
| 1377940_at | BF398271 | family with sequence similarity 101, member B | Fam101b | 4,77 | 7,37E-05 |
| 1388485_at | BG380414 | chemokine (C-X-C motif) ligand 14 | Cxcl14 | 4,77 | 3,63E-03 |
| 1376892_at | AI102061 | glutamate receptor, ionotrophic, AMPA 3 | Gria3 | 4,70 | 4,99E-04 |
| 1380250_at | AI013978 | sushi, nidogen and EGF-like domains 1 | Sned1 | 4,68 | 1,36E-05 |
| 1370155_at | BM388837 | collagen, type I, alpha 2 | Col1a2 | 4,66 | 1,68E-06 |
| 1372518_at | AI176918 | fibulin 1 | Fbln1 | 4,64 | 1,31E-05 |
| 1376799_a_at | AA925924 | cytokine receptor-like factor 1 | Crlf1 | 4,62 | 1,20E-04 |
| 1390860_at | AI230709 | insulin-like growth factor 2 mRNA binding protein 3 | Igf2bp3 | 4,60 | 8,43E-06 |
| 1381504_at | AI639412 | asporin | Aspn | 4,60 | 6,71E-05 |
| 1388618_at | BM389302 | nidogen 2 | Nid2 | 4,59 | 3,07E-06 |
| 1368000_at | NM_016994 | complement component 3 | C3 | 4,58 | 2,59E-06 |
| 1388347_at | AI233210 | lymphocyte antigen 6 complex, locus E | Ly6e | 4,57 | 5,10E-07 |
| 1387886_at | AI011747 | proline/arginine-rich end leucine-rich repeat protein | Prelp | 4,56 | 1,73E-04 |
| 1378168_at | BG377391 | family with sequence similarity 101, member B | Fam101b | 4,54 | 2,10E-04 |
| 1372647_at | BM388957 | proline arginine-rich end leucine-rich repeat protein-like | LOC100363743 | 4,46 | 2,93E-05 |
| 1387121_a_at | NM_133583 | N-myc downstream regulated gene 2 | Ndrg2 | 4,46 | 3,63E-05 |
| 1368590_at | NM_080776 | matrix metallopeptidase 16 | Mmp16 | 4,44 | 2,20E-04 |
| 1376047_at | BI285321 | 3'-phosphoadenosine 5'-phosphosulfate synthase 2 | Papss2 | 4,43 | 6,17E-05 |
| 1379885_at | BF282998 | flavin containing monooxygenase 4 | Fmo4 | 4,41 | 1,08E-06 |
| 1368103_at | NM_053502 | ATP-binding cassette, sub-family G (WHITE), member 1 | Abcg1 | 4,39 | 4,92E-04 |
| 1371298_at | BF284168 | H19, imprinted maternally expressed transcript | H19 | 4,39 | 5,48E-06 |
| 1388985_at | AI012869 | hypothetical LOC100361467 | LOC100361467 | 4,36 | 2,12E-03 |
| 1377853_at | AI599177 | teashirt zinc finger homeobox 3 | Tshz3 | 4,33 | 2,36E-05 |
| 1370972_x_at | BI300597 | RT1 class I, locus CE5 | RT1-CE5 | 4,32 | 7,28E-07 |
| 1373223_at | AI101361 | family with sequence similarity 171, member B | Fam171b | 4,32 | 1,38E-04 |
| 1377457_a_at | AA850618 | sortilin-related receptor, LDLR class A repeats-containing | Sorl1 | 4,28 | 3,10E-06 |
| 1387122_at | NM_012760 | pleiomorphic adenoma gene-like 1 | Plagl1 | 4,26 | 1,84E-05 |
| 1378163_at | AA817956 | gremlin 2, cysteine knot superfamily, homolog (Xenopus laevis) | Grem2 | 4,25 | 4,77E-05 |
| 1387011_at | NM_130741 | lipocalin 2 | Lcn2 | 4,24 | 6,14E-04 |
| 1368097_a_at | NM_053865 | reticulon 1 | Rtn1 | 4,22 | 4,46E-04 |
| 1379894_at | AI501165 | similar to 3632451O06Rik protein | RGD1310110 | 4,21 | 2,54E-04 |
| 1370218_at | AA848319 | lactate dehydrogenase B | Ldhb | 4,16 | 2,67E-05 |
| 1393026_at | BI289531 | ribonuclease, RNase A family, 1 (pancreatic) | Rnase1 | 4,15 | 1,02E-05 |
| 1370429_at | L40362 | RT1 class Ib, locus EC2 | RT1-EC2 | 4,14 | 6,42E-05 |
| 1388670_at | BI286860 | Kinesin family member 1A | Kif1a | 4,14 | 2,30E-04 |
| 1376908_at | AW531805 | interferon-induced protein with tetratricopeptide repeats 3 | Ifit3 | 4,13 | 2,25E-06 |
| 1380964_at | BF396607 | dystrobrevin alpha | Dtna | 4,12 | 2,53E-04 |
| 1385519_at | BE105678 | runt-related transcription factor 1; translocated to, 1 (cyclin D-related) | Runx1t1 | 4,09 | 9,44E-06 |
| 1368683_at | NM_133306 | oxidized low density lipoprotein (lectin-like) receptor 1 | Olr1 | 4,08 | 3,50E-05 |
| 1394946_at | BI289481 | hypothetical LOC292199 | LOC292199 | 4,07 | 1,36E-04 |
| 1393933_at | AW144823 | sortilin-related receptor, LDLR class A repeats-containing | Sorl1 | 4,07 | 4,78E-05 |
| 1387059_at | NM_019362 | serine/threonine kinase 39, STE20/SPS1 homolog (yeast) | Stk39 | 4,05 | 8,73E-08 |
| 1380442_at | AI235507 | Homeobox C9 | Hoxc9 | 4,04 | 5,18E-04 |
| 1390426_at | BF389398 | Notch homolog 1, translocation-associated (Drosophila) | Notch1 | 4,01 | 1,11E-03 |
| 1387316_at | NM_030845 | chemokine (C-X-C motif) ligand 1 (melanoma growth stimulating activity, alpha) | Cxcl1 | 4,00 | 1,94E-06 |
| 1382814_at | AW521702 | odz, odd Oz/ten-m homolog 3 (Drosophila) | Odz3 | 3,98 | 4,53E-06 |
| 1368672_at | NM_019168 | arginase type II | Arg2 | 3,97 | 1,34E-04 |
| 1382351_at | AI069972 | GTP binding protein (gene overexpressed in skeletal muscle) | Gem | 3,94 | 1,03E-05 |
| 1370834_at | AF177430 | heparan sulfate (glucosamine) 3-O-sulfotransferase 1 | Hs3st1 | 3,93 | 4,58E-05 |
| 1398484_at | AI070306 | similar to TBC1 domain family, member 8 (with GRAM domain); vascular Rab-GAP/TBC-containing | RGD1308221 | 3,92 | 3,15E-04 |
| 1369151_at | NM_053744 | delta-like 1 homolog (Drosophila) | Dlk1 | 3,91 | 8,44E-04 |
| 1383280_at | BG669130 | Transmembrane protein 56 | Tmem56 | 3,90 | 8,22E-05 |
| 1371349_at | AI598402 | collagen, type VI, alpha 1 | Col6a1 | 3,89 | 4,46E-05 |
| 1387259_at | AF097593 | cadherin 2 | Cdh2 | 3,88 | 1,15E-03 |
| 1367896_at | AB030829 | carbonic anhydrase 3 | Car3 | 3,87 | 1,52E-04 |
| 1368512_a_at | AF214568 | glutamyl aminopeptidase | Enpep | 3,87 | 1,76E-05 |
| 1368883_at | NM_030868 | nephroblastoma overexpressed gene | Nov | 3,87 | 3,95E-06 |
| 1393030_at | BE115641 | hypothetical protein LOC683963 /// similar to Myosin-9B (Myosin IXb) (Unconventional myosin-9b) /// hypothetical protein LOC691692 | LOC683963 /// LOC691401 /// LOC691692 | 3,84 | 1,34E-04 |
| 1370517_at | U18772 | neuronal pentraxin 1 | Nptx1 | 3,83 | 2,67E-03 |
| 1372084_at | AI104546 | protein tyrosine phosphatase 4a3 | Ptp4a3 | 3,81 | 3,43E-05 |
| 1387283_at | NM_134350 | myxovirus (influenza virus) resistance 2 | Mx2 | 3,79 | 3,43E-04 |
| 1371210_s_at | AJ276126 | RT1 class I, locus CE5 /// RT1 class Ib, locus EC2 | RT1-CE5 /// RT1-EC2 | 3,78 | 1,52E-04 |
| 1373977_at | BE108253 | Kinesin family member 5C | Kif5c | 3,78 | 7,60E-05 |
| 1368771_at | NM_134378 | sulfatase 1 | Sulf1 | 3,77 | 7,18E-05 |
| 1370371_a_at | U23056 | carcinoembryonic antigen-related cell adhesion molecule 1 (biliary glycoprotein) /// carcinoembryonic antigen-related cell adhesion molecule 10 | Ceacam1 /// Ceacam10 | 3,75 | 6,13E-06 |
| 1369008_a_at | NM_053573 | olfactomedin 1 | Olfm1 | 3,73 | 7,79E-04 |
| 1373427_at | BI288816 | Ras-related GTP binding D | Rragd | 3,73 | 5,53E-04 |
| 1385227_at | BF398245 | trichorhinophalangeal syndrome I homolog (human) | Trps1 | 3,72 | 1,96E-04 |
| 1384312_at | BF543574 | iroquois homeobox 1 | Irx1 | 3,72 | 2,82E-05 |
| 1386965_at | NM_012598 | lipoprotein lipase | Lpl | 3,72 | 3,28E-04 |
| 1382874_at | BI289407 | N-acetylneuraminate pyruvate lyase | Npl | 3,72 | 1,69E-04 |
| 1368945_at | NM_017178 | bone morphogenetic protein 2 | Bmp2 | 3,69 | 6,12E-04 |
| 1392550_at | BE111725 | Dapper, antagonist of beta-catenin, homolog 1 (Xenopus laevis) | Dact1 | 3,69 | 2,45E-05 |
| 1382201_at | AA926250 | unc-5 homolog C (C. elegans) | Unc5c | 3,69 | 8,42E-04 |
| 1387389_at | NM_020100 | receptor (G protein-coupled) activity modifying protein 3 | Ramp3 | 3,68 | 1,38E-05 |
| 1376259_at | AI171093 | protein kinase C, theta | Prkcq | 3,66 | 5,71E-05 |
| 1376775_at | AI059078 | proline rich 5 like | Prr5l | 3,66 | 3,32E-05 |
| 1385444_at | AI071994 | Dickkopf homolog 2 (Xenopus laevis) | Dkk2 | 3,65 | 1,39E-04 |
| 1391075_at | AI179271 | regulator of G-protein signaling 17 | Rgs17 | 3,63 | 8,38E-04 |
| 1375967_a_at | AI172067 | dual specificity phosphatase 22 | Dusp22 | 3,63 | 5,70E-05 |
| 1371491_at | BM390614 | Notch homolog 1, translocation-associated (Drosophila) | Notch1 | 3,62 | 6,09E-05 |
| 1383205_at | BI288833 | dapper, antagonist of beta-catenin, homolog 2 (Xenopus laevis) | Dact2 | 3,62 | 1,44E-04 |
| 1389193_at | BM388083 | sortilin-related VPS10 domain containing receptor 2 | Sorcs2 | 3,61 | 6,62E-07 |
| 1387294_at | NM_054011 | SH3-domain binding protein 5 (BTK-associated) | Sh3bp5 | 3,61 | 1,72E-06 |
| 1367814_at | M14137 | ATPase, Na+/K+ transporting, beta 1 polypeptide | Atp1b1 | 3,61 | 3,85E-06 |
| 1367786_at | NM_080767 | proteasome (prosome, macropain) subunit, beta type 8 (large multifunctional peptidase 7) | Psmb8 | 3,60 | 7,87E-05 |
| 1372423_at | BI286396 | PERP, TP53 apoptosis effector | Perp | 3,58 | 1,98E-06 |
| 1379390_at | AA891414 | ST6 (alpha-N-acetyl-neuraminyl-2,3-beta-galactosyl-1,3)-N-acetylgalactosaminide alpha-2,6-sialyltransferase 2 | St6galnac2 | 3,58 | 2,94E-05 |
| 1368021_at | NM_130780 | alcohol dehydrogenase 1 (class I) | Adh1 | 3,57 | 4,25E-05 |
| 1389164_at | BI295026 | hect domain and RLD 3 | Herc3 | 3,54 | 3,89E-05 |
| 1374778_at | AI409046 | cathepsin C | Ctsc | 3,53 | 4,55E-05 |
| 1371369_at | BI287851 | collagen, type VI, alpha 2 | Col6a2 | 3,53 | 4,41E-04 |
| 1385751_at | BF408413 | thrombospondin 2 | Thbs2 | 3,51 | 8,96E-06 |
| 1367919_at | NM_053322 | nucleoporin 210 | Nup210 | 3,50 | 1,63E-05 |
| 1394681_at | AW522526 | aldo-keto reductase family 1, member C-like 1 | Akr1cl1 | 3,50 | 8,78E-06 |
| 1382314_at | BE096523 | ISG15 ubiquitin-like modifier | Isg15 | 3,50 | 1,22E-04 |
| 1377239_at | BM386169 | amyloid beta (A4) precursor protein-binding, family B, member 1 interacting protein | Apbb1ip | 3,50 | 5,45E-04 |
| 1393547_at | BE101549 | ring finger protein 182 | Rnf182 | 3,48 | 1,64E-03 |
| 1376051_at | BI293393 | crystallin, lambda 1 | Cryl1 | 3,46 | 3,87E-07 |
| 1368914_at | NM_017325 | runt-related transcription factor 1 | Runx1 | 3,46 | 3,76E-06 |
| 1391146_at | BE111632 | cadherin 11 | Cdh11 | 3,45 | 1,95E-05 |
| 1371232_a_at | AF084544 | versican | Vcan | 3,44 | 1,31E-04 |
| 1393943_at | AI716693 | Matrilin 3 | Matn3 | 3,40 | 4,47E-05 |
| 1385229_at | AW524146 | protocadherin 20 | Pcdh20 | 3,39 | 3,46E-05 |
| 1387439_at | NM_022955 | multiple EGF-like-domains 6 | Megf6 | 3,39 | 2,26E-05 |
| 1371194_at | AF159103 | tumor necrosis factor alpha induced protein 6 | Tnfaip6 | 3,39 | 7,15E-05 |
| 1381556_at | BI303853 | DEAD (Asp-Glu-Ala-Asp) box polypeptide 60 | Ddx60 | 3,37 | 3,39E-04 |
| 1374006_at | BI295878 | kynurenine aminotransferase III /// kynurenine aminotransferase III-like | Kat3 /// LOC100361841 | 3,37 | 1,37E-03 |
| 1368564_at | NM_053427 | solute carrier family 17 (sodium-dependent inorganic phosphate cotransporter), member 6 | Slc17a6 | 3,36 | 1,47E-04 |
| 1394786_at | AW526631 | sortilin-related receptor, LDLR class A repeats-containing | Sorl1 | 3,35 | 6,72E-05 |
| 1391575_at | BG380566 | hyaluronan and proteoglycan link protein 4 | Hapln4 | 3,34 | 2,91E-05 |
| 1395721_at | AW917486 | 3'-phosphoadenosine 5'-phosphosulfate synthase 2 | Papss2 | 3,34 | 8,23E-04 |
| 1376693_at | AA998964 | similar to OEF2 | RGD1563091 | 3,31 | 3,39E-05 |
| 1382230_at | BI296330 | kelch domain containing 8A | Klhdc8a | 3,30 | 2,43E-04 |
| 1373410_at | BE104219 | myocyte enhancer factor 2C | Mef2c | 3,30 | 1,69E-05 |
| 1386995_at | BI288701 | BTG family, member 2 | Btg2 | 3,30 | 2,86E-06 |
| 1398246_s_at | NM_053843 | Fc fragment of IgG, low affinity IIa, receptor (CD32) /// Fc gamma receptor II beta | Fcgr2a /// LOC498276 | 3,30 | 3,31E-04 |
| 1368223_at | NM_024400 | ADAM metallopeptidase with thrombospondin type 1 motif, 1 | Adamts1 | 3,29 | 1,06E-05 |
| 1378369_at | BE107150 | dapper, antagonist of beta-catenin, homolog 1 (Xenopus laevis) | Dact1 | 3,29 | 1,26E-04 |
| 1370037_at | NM_031826 | fibrillin 2 | Fbn2 | 3,29 | 6,13E-05 |
| 1374649_at | BG380629 | RAS guanyl releasing protein 2 (calcium and DAG-regulated) | Rasgrp2 | 3,28 | 6,94E-06 |
| 1373514_at | AA899109 | ring finger protein 213 | Rnf213 | 3,28 | 2,03E-04 |
| 1382274_at | AA819288 | retinoic acid receptor responder (tazarotene induced) 1 | Rarres1 | 3,28 | 8,54E-04 |
| 1374970_at | BF392911 | wingless-type MMTV integration site family, member 5B | Wnt5b | 3,26 | 8,88E-04 |
| 1377369_at | BF419070 | cytochrome b reductase 1 | Cybrd1 | 3,26 | 8,76E-06 |
| 1368202_a_at | NM_024159 | disabled homolog 2 (Drosophila) | Dab2 | 3,26 | 1,94E-06 |
| 1376197_at | AW251860 | transcription factor 7, T-cell specific | Tcf7 | 3,25 | 1,02E-04 |
| 1388657_at | AW253923 | dachsous 1 (Drosophila) | Dchs1 | 3,23 | 9,45E-04 |
| 1370606_at | U22830 | purinergic receptor P2Y, G-protein coupled, 1 | P2ry1 | 3,23 | 1,55E-03 |
| 1396614_at | BF396545 | secreted frizzled-related protein 2 | Sfrp2 | 3,22 | 1,85E-04 |
| 1395316_at | H32543 | melanoma antigen, family H, 1 | Mageh1 | 3,22 | 1,17E-05 |
| 1367568_a_at | NM_012862 | matrix Gla protein | Mgp | 3,22 | 9,57E-07 |
| 1392915_at | BM389291 | collagen, type XI, alpha 1 | Col11a1 | 3,22 | 1,21E-04 |
| 1374337_at | AI408954 | ring finger protein 213 | Rnf213 | 3,21 | 2,50E-05 |
| 1370583_s_at | AY082609 | ATP-binding cassette, sub-family B (MDR/TAP), member 1A /// ATP-binding cassette, sub-family B (MDR/TAP), member 1B | Abcb1a /// Abcb1b | 3,20 | 9,06E-07 |
| 1376937_at | BM389685 | similar to 4631422O05Rik protein | RGD1565927 | 3,20 | 6,58E-06 |
| 1398272_at | NM_022860 | beta-1,4-N-acetyl-galactosaminyl transferase 1 | B4galnt1 | 3,19 | 8,82E-05 |
| 1389659_at | AI230591 | cytotoxic T lymphocyte-associated protein 2 alpha | Ctla2a | 3,19 | 2,33E-03 |
| 1391200_at | AW530290 | sprouty homolog 3 (Drosophila) | Spry3 | 3,18 | 2,51E-06 |
| 1368187_at | NM_133298 | glycoprotein (transmembrane) nmb | Gpnmb | 3,17 | 1,84E-07 |
| 1395986_at | BF391439 | slit homolog 2 (Drosophila) | Slit2 | 3,16 | 2,43E-05 |
| 1370642_s_at | BM389426 | platelet derived growth factor receptor, beta polypeptide | Pdgfrb | 3,16 | 1,81E-04 |
| 1374796_at | AI413058 | sarcoglycan, beta (dystrophin-associated glycoprotein) | Sgcb | 3,16 | 9,12E-06 |
| 1379935_at | BF419899 | chemokine (C-C motif) ligand 7 | Ccl7 | 3,15 | 2,01E-04 |
| 1377643_at | BI291489 | homeo box D10 | Hoxd10 | 3,15 | 7,44E-03 |
| 1371186_at | AA955091 | integrin, alpha 6 | Itga6 | 3,15 | 2,32E-03 |
| 1393558_at | AI137931 | Integrin, alpha 6 | Itga6 | 3,15 | 5,38E-04 |
| 1374207_at | BI275292 | angiopoietin 2 | Angpt2 | 3,13 | 2,84E-05 |
| 1371015_at | X52711 | myxovirus (influenza virus) resistance 1 | Mx1 | 3,13 | 2,28E-04 |
| 1374594_at | AI599133 | similar to RIKEN cDNA 1600029D21 | LOC363060 | 3,13 | 2,96E-05 |
| 1375428_at | BE099979 | cellular repressor of E1A-stimulated genes 1 | Creg1 | 3,13 | 2,05E-05 |
| 1373036_at | BF283621 | mCG114897-like | LOC100363987 | 3,12 | 3,69E-06 |
| 1379285_at | AA819788 | receptor (chemosensory) transporter protein 4 | Rtp4 | 3,12 | 6,97E-05 |
| 1383794_at | AI575277 | similar to RIKEN cDNA A930038C07 | RGD1311080 | 3,11 | 3,62E-04 |
| 1388809_at | BM389498 | sphingomyelin phosphodiesterase, acid-like 3A | Smpdl3a | 3,11 | 7,21E-04 |
| 1384202_at | BI287326 | tescalcin | Tesc | 3,10 | 4,73E-04 |
| 1381968_at | AI029175 | cellular repressor of E1A-stimulated genes 1 | Creg1 | 3,10 | 1,23E-07 |
| 1388821_at | AI010430 | tribbles homolog 2 (Drosophila) | Trib2 | 3,10 | 1,79E-04 |
| 1378144_at | BI296384 | KN motif and ankyrin repeat domains 4 | Kank4 | 3,09 | 1,20E-04 |
| 1373829_at | AI412658 | fibroblast growth factor receptor 2 | Fgfr2 | 3,09 | 1,45E-04 |
| 1367866_at | NM_019153 | fibulin 5 | Fbln5 | 3,09 | 5,31E-05 |
| 1383322_at | BG375198 | RAS-like family 11 member B | Rasl11b | 3,09 | 6,23E-07 |
| 1378196_at | BI290895 | solute carrier family 43, member 1 | Slc43a1 | 3,08 | 1,44E-04 |
| 1368840_at | NM_134390 | transmembrane protein 176B | Tmem176b | 3,07 | 6,01E-04 |
| 1370342_at | AF385402 | potassium channel, subfamily K, member 2 | Kcnk2 | 3,07 | 2,96E-05 |
| 1379911_at | BI281823 | death associated protein kinase 1 | Dapk1 | 3,06 | 2,42E-04 |
| 1387147_at | NM_133536 | RAB3C, member RAS oncogene family | Rab3c | 3,05 | 1,96E-04 |
| 1390795_at | AI029455 | tocopherol (alpha) transfer protein | Ttpa | 3,04 | 4,57E-04 |
| 1368931_at | NM_031238 | SH3-domain GRB2-like 3 | Sh3gl3 | 3,04 | 2,69E-04 |
| 1397335_at | AW532165 | sema domain, immunoglobulin domain (Ig), short basic domain, secreted, (semaphorin) 3D | Sema3d | 3,04 | 6,58E-04 |
| 1370503_s_at | AB032828 | erythrocyte protein band 4.1-like 3 | Epb4.1l3 | 3,04 | 5,06E-06 |
| 1369793_a_at | AB035507 | melanoma cell adhesion molecule | Mcam | 3,03 | 1,35E-04 |
| 1371257_at | BF386897 | RAR-related orphan receptor B | Rorb | 3,02 | 9,00E-05 |
| 1367850_at | NM_053843 | Fc fragment of IgG, low affinity IIa, receptor (CD32) /// Low affinity immunoglobulin gamma Fc region receptor III-like /// Fc gamma receptor II beta | Fcgr2a /// LOC100362543 /// LOC498276 | 3,01 | 3,44E-05 |
| 1393454_at | BF558981 | protocadherin 17 | Pcdh17 | 3,01 | 4,51E-03 |
| 1373140_at | AA851740 | interleukin 6 signal transducer | Il6st | 3,01 | 1,77E-05 |
| 1393737_at | AI044560 | gastrokine 2 | Gkn2 | 3,01 | 1,15E-03 |
| 1369686_at | U78857 | doublecortin-like kinase 1 | Dclk1 | 3,00 | 8,75E-05 |
| 1397367_at | AI501213 | Hypothetical protein LOC689663 | LOC689663 | 3,00 | 6,28E-04 |
| 1368418_a_at | AF202115 | ceruloplasmin | Cp | 3,00 | 5,69E-06 |
| 1383766_at | BG663338 | sarcoglycan, beta (dystrophin-associated glycoprotein) | Sgcb | 3,00 | 5,18E-06 |
| 1379748_at | AA819629 | Interferon-induced protein 44-like | Ifi44l | 3,00 | 8,84E-04 |
| 1388152_at | BG374290 | microtubule-associated protein 2 | Map2 | 2,97 | 2,06E-05 |
| 1370830_at | M37394 | epidermal growth factor receptor | Egfr | 2,97 | 2,89E-04 |
| 1369338_at | NM_022188 | roundabout homolog 1 (Drosophila) | Robo1 | 2,97 | 8,15E-04 |
| 1385382_at | BI294158 | a disintegrin and metallopeptidase domain 19 (meltrin beta) | Adam19 | 2,97 | 1,18E-03 |
| 1387571_at | NM_031130 | nuclear receptor subfamily 2, group F, member 1 | Nr2f1 | 2,96 | 5,35E-03 |
| 1377034_at | BF411331 | serine (or cysteine) proteinase inhibitor, clade B, member 1a | Serpinb1a | 2,96 | 1,21E-03 |
| 1389020_at | BM389149 | similar to immunoglobulin superfamily containing leucine-rich repeat | LOC686539 | 2,95 | 2,77E-04 |
| 1387726_at | NM_023963 | caudal type homeo box 2 | Cdx2 | 2,95 | 6,77E-04 |
| 1383593_at | BE113211 | Transmembrane protein 56 | Tmem56 | 2,94 | 1,75E-05 |
| 1373188_at | AI137995 | sodium channel, voltage-gated, type IV, beta | Scn4b | 2,94 | 1,08E-04 |
| 1376657_at | BE117767 | cell adhesion molecule 1 | Cadm1 | 2,93 | 4,18E-04 |
| 1389151_at | AI103440 | a disintegrin and metallopeptidase domain 19 (meltrin beta) | Adam19 | 2,92 | 3,50E-05 |
| 1368738_at | D11354 | cytochrome P450, family 11, subfamily b, polypeptide 1 | Cyp11b1 | 2,92 | 4,00E-03 |
| 1373912_at | AI170859 | ectonucleotide pyrophosphatase/phosphodiesterase 4 | Enpp4 | 2,91 | 1,74E-05 |
| 1367784_a_at | AF314657 | clusterin | Clu | 2,90 | 5,74E-09 |
| 1384023_at | AI229556 | spindlin family, member 2A | Spin2a | 2,90 | 5,57E-05 |
| 1382387_at | BF288508 | anoctamin 1, calcium activated chloride channel | Ano1 | 2,90 | 3,81E-04 |
| 1378402_at | BE109781 | grainyhead-like 3 (Drosophila) | Grhl3 | 2,90 | 9,59E-04 |
| 1386937_at | AI232036 | ATPase, Na+/K+ transporting, beta 1 polypeptide | Atp1b1 | 2,89 | 6,88E-05 |
| 1392888_at | AI071251 | glypican 4 | Gpc4 | 2,89 | 9,78E-06 |
| 1368419_at | AF202115 | ceruloplasmin | Cp | 2,89 | 1,28E-04 |
| 1388936_at | BI296340 | cadherin 11 | Cdh11 | 2,89 | 2,93E-06 |
| 1373578_at | BM386413 | tripartite motif-containing 2 | Trim2 | 2,89 | 4,53E-04 |
| 1387659_at | AF245172 | guanine deaminase | Gda | 2,89 | 1,38E-04 |
| 1369973_at | NM_017154 | xanthine dehydrogenase | Xdh | 2,88 | 1,32E-05 |
| 1368322_at | NM_012880 | superoxide dismutase 3, extracellular | Sod3 | 2,88 | 1,27E-07 |
| 1368105_at | AI228231 | tetraspanin 2 | Tspan2 | 2,88 | 1,54E-03 |
| 1391022_at | BE101834 | laminin, beta 3 | Lamb3 | 2,88 | 7,65E-06 |
| 1376013_at | BF282700 | tetratricopeptide repeat domain 9 | Ttc9 | 2,87 | 4,61E-05 |
| 1398370_at | AW522471 | adenosine deaminase, RNA-specific, B1 | Adarb1 | 2,87 | 3,74E-04 |
| 1374939_at | BE112927 | cytoplasmic FMR1 interacting protein 2 | Cyfip2 | 2,87 | 5,34E-04 |
| 1374933_at | BI277043 | melanoma cell adhesion molecule | Mcam | 2,87 | 1,15E-05 |
| 1370341_at | AF019973 | enolase 2, gamma, neuronal | Eno2 | 2,86 | 6,57E-04 |
| 1381548_at | BF397673 | golgi integral membrane protein 4 | Golim4 | 2,86 | 7,12E-05 |
| 1398866_at | AF255614 | membrane associated guanylate kinase, WW and PDZ domain containing 3 | Magi3 | 2,86 | 1,48E-04 |
| 1389177_at | AI598971 | PERP, TP53 apoptosis effector | Perp | 2,85 | 8,40E-05 |
| 1384866_at | AI070096 | ectonucleoside triphosphate diphosphohydrolase 3 | Entpd3 | 2,85 | 1,00E-03 |
| 1373258_at | AI169359 | cathepsin F | Ctsf | 2,85 | 9,86E-05 |
| 1370609_a_at | X97445 | solute carrier family 16, member 7 (monocarboxylic acid transporter 2) | Slc16a7 | 2,84 | 9,78E-05 |
| 1382272_at | BE349785 | angiotensin II receptor-associated protein | Agtrap | 2,83 | 5,87E-04 |
| 1368174_at | NM_019371 | EGL nine homolog 3 (C. elegans) | Egln3 | 2,82 | 4,17E-06 |
| 1391279_at | AI112564 | Scinderin | Scin | 2,82 | 5,45E-05 |
| 1384580_at | AI045191 | complement component 6 | C6 | 2,81 | 6,25E-06 |
| 1387498_a_at | S54008 | Fibroblast growth factor receptor 1 | Fgfr1 | 2,81 | 4,63E-04 |
| 1387669_a_at | NM_012844 | epoxide hydrolase 1, microsomal | Ephx1 | 2,79 | 5,60E-05 |
| 1370157_at | BI290034 | phospholamban | Pln | 2,79 | 4,43E-05 |
| 1372682_at | BI289460 | similar to RIKEN cDNA 2810432L12 | RGD1307218 | 2,79 | 3,44E-03 |
| 1380596_at | BE098869 | desmoglein 2 | Dsg2 | 2,79 | 3,15E-06 |
| 1383489_at | BG663025 | interleukin 6 signal transducer | Il6st | 2,78 | 4,25E-05 |
| 1373546_at | AI409922 | ATPase, class VI, type 11A | Atp11a | 2,78 | 1,91E-04 |
| 1384506_at | H33706 | unc-5 homolog C (C. elegans) | Unc5c | 2,76 | 5,32E-04 |
| 1386344_at | BG662519 | Ankylosis, progressive homolog (mouse) | Ankh | 2,76 | 7,85E-04 |
| 1373865_at | BF281248 | synaptosomal-associated protein 91 | Snap91 | 2,75 | 2,94E-04 |
| 1383673_at | BI288925 | nucleosome assembly protein 1-like 2 | Nap1l2 | 2,75 | 3,17E-03 |
| 1388212_a_at | AJ243974 | RT1 class Ib, locus S3 | RT1-S3 | 2,75 | 4,13E-08 |
| 1372989_at | BI296586 | zinc finger, DHHC-type containing 14 | Zdhhc14 | 2,74 | 9,66E-05 |
| 1388213_a_at | AJ243973 | RT1 class Ib, locus S3 | RT1-S3 | 2,73 | 4,21E-05 |
| 1393706_at | BE109939 | six transmembrane epithelial antigen of the prostate 1 | Steap1 | 2,73 | 1,47E-05 |
| 1369895_s_at | AF109393 | podocalyxin-like | Podxl | 2,73 | 3,82E-05 |
| 1377497_at | BF419319 | 2'-5'-oligoadenylate synthetase-like | Oasl | 2,71 | 2,89E-05 |
| 1397184_at | AI574688 | hypothetical LOC292199 | LOC292199 | 2,70 | 1,14E-04 |
| 1387010_s_at | NM_017288 | sodium channel, voltage-gated, type I, beta | Scn1b | 2,69 | 5,89E-04 |
| 1383662_at | AI043759 | hypothetical protein LOC500956 | LOC500956 | 2,69 | 4,34E-05 |
| 1370810_at | L09752 | cyclin D2 | Ccnd2 | 2,68 | 1,19E-04 |
| 1371078_at | AI500830 | RT1 class Ib, locus EC2 | RT1-EC2 | 2,68 | 2,79E-04 |
| 1391169_at | BE120139 | transmembrane protein 117 | Tmem117 | 2,67 | 4,25E-04 |
| 1380866_at | AA817746 | similar to adenylate kinase 5 isoform 1 | LOC365985 | 2,67 | 6,67E-05 |
| 1382118_at | BE104676 | Antisense paternally expressed gene 3 | Apeg3 | 2,67 | 2,72E-03 |
| 1370957_at | BM383427 | interleukin 6 signal transducer | Il6st | 2,66 | 1,65E-05 |
| 1383564_at | BF411036 | interferon regulatory factor 7 | Irf7 | 2,66 | 1,04E-06 |
| 1368515_at | NM_053927 | erythrocyte protein band 4.1-like 3 | Epb4.1l3 | 2,66 | 1,45E-03 |
| 1373315_at | AI176425 | aryl hydrocarbon receptor nuclear translocator 2 | Arnt2 | 2,65 | 6,56E-05 |
| 1384392_at | BF397093 | cytochrome P450, family 26, subfamily b, polypeptide 1 | Cyp26b1 | 2,64 | 2,30E-05 |
| 1388647_at | AI233753 | similar to coiled-coil-helix-coiled-coil-helix domain containing 7 | LOC684258 | 2,63 | 1,46E-06 |
| 1376584_at | BE116408 | protein phosphatase 1 (formerly 2C)-like | Ppm1l | 2,62 | 1,12E-04 |
| 1391935_at | AI575608 | eukaryotic translation initiation factor 4E member 3 | Eif4e3 | 2,62 | 3,55E-06 |
| 1382685_at | AI101660 | slit homolog 2 (Drosophila) | Slit2 | 2,61 | 1,85E-05 |
| 1370927_at | BE108345 | collagen, type XII, alpha 1 | Col12a1 | 2,61 | 1,70E-05 |
| 1387753_s_at | NM_139084 | membrane associated guanylate kinase, WW and PDZ domain containing 3 | Magi3 | 2,60 | 1,42E-04 |
| 1376481_at | BF416285 | a disintegrin-like and metalloprotease (reprolysin type) with thrombospondin type 1 motif, 9 | Adamts9 | 2,60 | 3,33E-04 |
| 1373266_at | BG380826 | family with sequence similarity 107, member A /// downregulated in renal cell carcinoma-like | Fam107a /// LOC100364831 | 2,60 | 2,18E-04 |
| 1380317_at | BF402765 | cadherin 10 | Cdh10 | 2,60 | 2,88E-03 |
| 1368940_at | NM_017255 | purinergic receptor P2Y, G-protein coupled, 2 | P2ry2 | 2,59 | 3,89E-04 |
| 1370374_at | AF335281 | STEAP family member 3 | Steap3 | 2,59 | 5,52E-05 |
| 1390776_at | AI030203 | iroquois homeobox 3 | Irx3 | 2,57 | 1,56E-04 |
| 1383309_at | BF522030 | ST3 beta-galactoside alpha-2,3-sialyltransferase 6 | St3gal6 | 2,57 | 6,25E-05 |
| 1398482_at | AI411774 | B-cell CLL/lymphoma 3 | Bcl3 | 2,56 | 4,98E-05 |
| 1389210_at | BE109711 | lymphocyte cytosolic protein 1 | Lcp1 | 2,56 | 6,26E-04 |
| 1371951_at | AA800031 | four and a half LIM domains 2 | Fhl2 | 2,56 | 9,62E-05 |
| 1374616_at | BM384311 | platelet-derived growth factor receptor-like | Pdgfrl | 2,56 | 1,03E-05 |
| 1388078_a_at | AB049451 | amiloride-sensitive cation channel 2, neuronal | Accn2 | 2,54 | 1,83E-04 |
| 1398615_at | BG379594 | GTPase activating Rap/RanGAP domain-like 4 | Garnl4 | 2,54 | 1,06E-05 |
| 1383219_at | BE109305 | CAP-GLY domain containing linker protein family, member 4 | Clip4 | 2,54 | 5,00E-03 |
| 1398378_at | AI231779 | glutathione S-transferase kappa 1 | Gstk1 | 2,54 | 5,26E-04 |
| 1387789_at | NM_133397 | v-ets erythroblastosis virus E26 oncogene homolog (avian) | Erg | 2,54 | 3,19E-04 |
| 1394570_at | BF284360 | X-linked Kx blood group (McLeod syndrome) homolog | Xk | 2,53 | 2,22E-04 |
| 1371071_at | BI284800 | guanine nucleotide binding protein (G protein), beta polypeptide 4 | Gnb4 | 2,53 | 2,06E-05 |
| 1367959_a_at | AF182949 | sodium channel, voltage-gated, type I, beta | Scn1b | 2,53 | 8,06E-05 |
| 1373803_a_at | AI170771 | growth hormone receptor | Ghr | 2,53 | 8,95E-04 |
| 1381976_at | BE103004 | kinesin family member 21A | Kif21a | 2,52 | 3,19E-04 |
| 1369654_at | NM_023991 | protein kinase, AMP-activated, alpha 2 catalytic subunit | Prkaa2 | 2,52 | 2,54E-04 |
| 1384781_at | AA965176 | zinc finger homeobox 4 | Zfhx4 | 2,51 | 1,86E-05 |
| 1389732_at | BM386323 | damage-regulated autophagy modulator | Dram | 2,51 | 2,31E-04 |
| 1369516_at | NM_022852 | pancreatic and duodenal homeobox 1 | Pdx1 | 2,51 | 2,00E-04 |
| 1392547_at | AI716211 | hypothetical LOC302884 | MGC105649 | 2,50 | 3,21E-05 |
| 1370956_at | BM390253 | decorin | Dcn | 2,50 | 5,74E-06 |
| 1392943_at | BG665934 | membrane bound O-acyltransferase domain containing 2 | Mboat2 | 2,50 | 9,20E-04 |
| 1384302_at | AI712791 | solute carrier family 6 (neurotransmitter transporter), member 17 | Slc6a17 | 2,49 | 2,04E-04 |
| 1370638_at | AF069525 | ankyrin 3, epithelial | Ank3 | 2,48 | 5,06E-05 |
| 1372895_at | AI169367 | similar to RIKEN cDNA 5730469M10 | RGD1309676 | 2,48 | 8,18E-06 |
| 1368280_at | NM_017097 | cathepsin C | Ctsc | 2,48 | 1,63E-05 |
| 1392996_at | BG668435 | cytoplasmic polyadenylation element binding protein 1 | Cpeb1 | 2,48 | 3,19E-04 |
| 1370072_at | NM_012608 | membrane metallo endopeptidase | Mme | 2,48 | 2,67E-04 |
| 1390710_x_at | AA850618 | sortilin-related receptor, LDLR class A repeats-containing | Sorl1 | 2,48 | 3,44E-03 |
| 1372447_at | BI275155 | Fibroblast growth factor receptor 1 | Fgfr1 | 2,48 | 1,75E-04 |
| 1372658_at | BG373779 | synemin, intermediate filament protein | Synm | 2,48 | 2,02E-04 |
| 1387099_at | NM_053838 | natriuretic peptide receptor B/guanylate cyclase B (atrionatriuretic peptide receptor B) | Npr2 | 2,47 | 3,83E-04 |
| 1389109_at | AI407351 | phosphatidylinositol-5-phosphate 4-kinase, type II, alpha | Pip4k2a | 2,47 | 3,74E-06 |
| 1392595_at | AI228656 | zinc finger protein 618 | Znf618 | 2,47 | 2,12E-05 |
| 1388712_at | BM389186 | cytohesin 3 | Cyth3 | 2,47 | 8,39E-06 |
| 1389095_at | BE110539 | biregional cell adhesion molecule-related/down-regulated by oncogenes (Cdon) binding protein | Boc | 2,47 | 1,86E-03 |
| 1398606_at | BM391878 | golgi integral membrane protein 4 | Golim4 | 2,46 | 6,75E-08 |
| 1372626_at | AI231999 | tumor protein D52-like 1 | Tpd52l1 | 2,46 | 9,92E-05 |
| 1390812_a_at | AI170076 | RAS-like, estrogen-regulated, growth-inhibitor | Rerg | 2,46 | 1,56E-03 |
| 1382084_at | AA850264 | Similar to EHM2 | RGD1562988 | 2,45 | 8,00E-05 |
| 1368671_at | NM_022524 | sushi-repeat-containing protein, X-linked | Srpx | 2,45 | 3,86E-04 |
| 1368207_at | NM_021909 | FXYD domain-containing ion transport regulator 5 | Fxyd5 | 2,45 | 6,58E-07 |
| 1378003_at | BM391860 | leucine rich repeat containing 8 family, member B | Lrrc8b | 2,45 | 4,63E-05 |
| 1384132_at | H31111 | cell adhesion molecule 1 | Cadm1 | 2,43 | 8,41E-04 |
| 1371123_x_at | AJ243973 | RT1 class Ib, locus S3 | RT1-S3 | 2,43 | 1,58E-05 |
| 1375959_at | AI007970 | naked cuticle homolog 1 (Drosophila) | Nkd1 | 2,43 | 4,20E-04 |
| 1394490_at | AI502114 | ATP-binding cassette, sub-family A (ABC1), member 1 | Abca1 | 2,43 | 9,17E-06 |
| 1372182_at | BM389769 | phosphofructokinase, platelet | Pfkp | 2,43 | 1,64E-06 |
| 1385051_at | BE108569 | guanylate binding protein 4 | Gbp4 | 2,42 | 7,02E-05 |
| 1392512_at | AW533007 | histone cluster 3, H2ba | Hist3h2ba | 2,42 | 3,28E-05 |
| 1372702_at | BI284972 | proline rich protein 2-like 1 | Prp2l1 | 2,42 | 1,16E-03 |
| 1376771_at | BF412303 | protein phosphatase 1 (formerly 2C)-like | Ppm1l | 2,42 | 7,30E-04 |
| 1371412_a_at | BE107450 | collagen, type VI, alpha 1 | Col6a1 | 2,41 | 1,09E-06 |
| 1369425_at | NM_138889 | cadherin 13 | Cdh13 | 2,41 | 1,71E-05 |
| 1371926_at | AI171807 | interleukin 6 signal transducer | Il6st | 2,41 | 9,39E-08 |
| 1382311_at | BM384466 | TRAF-interacting protein with forkhead-associated domain | Tifa | 2,40 | 2,19E-03 |
| 1370282_at | U44948 | cysteine and glycine-rich protein 2 | Csrp2 | 2,40 | 1,99E-05 |
| 1372000_at | AI180187 | neuroepithelial cell transforming 1 | Net1 | 2,40 | 5,58E-05 |
| 1371152_a_at | Z18877 | 2'-5' oligoadenylate synthetase 1A | Oas1a | 2,39 | 8,02E-04 |
| 1372325_at | BI303596 | elastin microfibril interfacer 1 | Emilin1 | 2,39 | 1,94E-04 |
| 1387274_at | NM_012943 | distal-less homeobox 5 | Dlx5 | 2,38 | 6,75E-03 |
| 1372980_at | AA819250 | tetraspanin 33 | Tspan33 | 2,38 | 6,54E-04 |
| 1394865_at | AI709592 | receptor (chemosensory) transporter protein 3 | Rtp3 | 2,37 | 3,48E-03 |
| 1388164_at | AF029241 | RT1 class Ib, locus S3 | RT1-S3 | 2,37 | 1,51E-04 |
| 1384044_at | AA964882 | sarcoglycan, beta (dystrophin-associated glycoprotein) | Sgcb | 2,37 | 6,05E-04 |
| 1370229_at | BG666709 | N-myc downstream regulated gene 4 | Ndrg4 | 2,37 | 4,67E-06 |
| 1389654_at | BM392070 | Plastin 1 (I isoform) | Pls1 | 2,37 | 7,41E-06 |
| 1375945_at | BG375285 | plexin domain containing 2 | Plxdc2 | 2,37 | 3,31E-03 |
| 1389734_x_at | BI282965 | RT1 class I, locus T24, gene 4 | RT1-T24-4 | 2,36 | 1,79E-06 |
| 1384509_s_at | BF558981 | protocadherin 17 | Pcdh17 | 2,36 | 1,44E-03 |
| 1377923_at | AA892818 | StAR-related lipid transfer (START) domain containing 8 | Stard8 | 2,36 | 2,37E-03 |
| 1390863_at | BF283298 | solute carrier family 19 (thiamine transporter), member 2 | Slc19a2 | 2,36 | 1,87E-05 |
| 1373368_at | BI279680 | procollagen C-endopeptidase enhancer 2 | PCOLCE2 | 2,36 | 3,06E-04 |
| 1384756_at | BF394311 | solute carrier family 43, member 2 | Slc43a2 | 2,36 | 4,06E-03 |
| 1388963_at | AI102087 | astrotactin 1 | Astn1 | 2,36 | 5,38E-04 |
| 1370907_at | M83143 | ST6 beta-galactosamide alpha-2,6-sialyltranferase 1 | St6gal1 | 2,36 | 7,03E-05 |
| 1387141_at | NM_023023 | dihydropyrimidinase-like 5 | Dpysl5 | 2,35 | 1,38E-03 |
| 1373345_at | AW523747 | adhesion molecule with Ig like domain 2 | Amigo2 | 2,34 | 7,48E-03 |
| 1388874_at | BE113032 | metastasis suppressor 1 | Mtss1 | 2,34 | 5,51E-05 |
| 1387407_at | NM_133402 | nucleosome assembly protein 1-like 3 | Nap1l3 | 2,34 | 7,61E-04 |
| 1376040_at | BI290044 | signal-induced proliferation-associated 1 like 2 | Sipa1l2 | 2,34 | 9,46E-05 |
| 1375719_s_at | BG381748 | cadherin 13 | Cdh13 | 2,34 | 3,31E-07 |
| 1384874_at | BF558516 | Similar to MIR-interacting saposin-like protein precursor (Transmembrane protein 4) (Putative secreted protein ZSIG9) | LOC685001 | 2,34 | 3,25E-04 |
| 1389662_at | AI406290 | WNK lysine deficient protein kinase 4 | Wnk4 | 2,33 | 7,03E-04 |
| 1387305_s_at | NM_012539 | cytochrome P450, family 11, subfamily b, polypeptide 1 /// cytochrome P450, family 11, subfamily b, polypeptide 2 | Cyp11b1 /// Cyp11b2 | 2,33 | 7,52E-04 |
| 1374417_at | BE113111 | nuclear receptor binding protein 2 | Nrbp2 | 2,33 | 1,24E-04 |
| 1367658_at | NM_021676 | SH3 and multiple ankyrin repeat domains 3 | Shank3 | 2,33 | 6,69E-05 |
| 1371006_at | BI274746 | jagged 2 | Jag2 | 2,33 | 2,19E-05 |
| 1392905_at | BF552733 | Guanine nucleotide binding protein (G protein), gamma 2 | Gng2 | 2,32 | 1,46E-05 |
| 1390396_at | AI101338 | eukaryotic translation initiation factor 4E member 3 | Eif4e3 | 2,32 | 7,67E-04 |
| 1387677_at | M84645 | POU class 3 homeobox 4 | Pou3f4 | 2,31 | 2,67E-04 |
| 1384180_at | BE118697 | interferon-induced protein with tetratricopeptide repeats 2 | Ifit2 | 2,31 | 8,89E-03 |
| 1382612_at | BF395928 | homeobox A9-like | Hoxa9l | 2,31 | 3,02E-03 |
| 1387241_at | NM_031696 | G-protein coupled receptor 88 | Gpr88 | 2,30 | 2,84E-03 |
| 1373011_at | BE109520 | family with sequence similarity 134, member B | Fam134b | 2,30 | 9,57E-05 |
| 1370459_at | AY007690 | alanine and arginine rich domain containing protein | Aard | 2,30 | 4,25E-04 |
| 1384469_at | AI502224 | ATP-binding cassette, sub-family A (ABC1), member 5 | Abca5 | 2,30 | 3,29E-04 |
| 1390050_at | BI288898 | similar to Golgi phosphoprotein 2 (Golgi membrane protein GP73) | LOC680692 | 2,30 | 2,23E-03 |
| 1393659_at | BE109193 | translocation associated membrane protein 1-like 1 | Tram1l1 | 2,30 | 2,54E-04 |
| 1375420_at | AI170535 | tumor protein p53 inducible protein 11 | Tp53i11 | 2,29 | 3,97E-04 |
| 1372724_at | BI284904 | glutamate receptor, ionotropic, N-methyl D-aspartate-associated protein 1 (glutamate binding) | Grina | 2,29 | 7,01E-05 |
| 1370570_at | AF016296 | neuropilin 1 | Nrp1 | 2,29 | 1,23E-04 |
| 1388836_at | AA799981 | protein kinase C, eta | Prkch | 2,29 | 1,68E-03 |
| 1375066_at | H33003 | similar to RIKEN cDNA 6330512M04 gene | RGD1563319 | 2,29 | 1,15E-03 |
| 1379783_at | AI113136 | hypothetical protein LOC686432 | LOC686432 | 2,28 | 7,59E-05 |
| 1383266_at | AW144660 | secreted frizzled-related protein 1 | Sfrp1 | 2,28 | 1,32E-03 |
| 1389653_at | AW526087 | protocadherin beta 9 | Pcdhb9 | 2,28 | 4,89E-04 |
| 1373272_at | AI009219 | pleckstrin homology domain containing, family A member 5 | Plekha5 | 2,28 | 3,86E-04 |
| 1388433_at | BI279605 | keratin 19 | Krt19 | 2,28 | 1,29E-04 |
| 1383241_at | BI292425 | complement component 1, r subcomponent | C1r | 2,28 | 2,24E-06 |
| 1388673_at | AI012109 | lymphocyte-specific protein 1 | Lsp1 | 2,27 | 2,89E-04 |
| 1370585_a_at | X04440 | protein kinase C, beta | Prkcb | 2,27 | 1,65E-03 |
| 1384540_at | BE101066 | leucine rich repeat and fibronectin type III domain containing 3 | Lrfn3 | 2,26 | 1,64E-04 |
| 1372734_at | AI408095 | small trans-membrane and glycosylated protein | Smagp | 2,25 | 2,88E-03 |
| 1372208_at | AA942959 | protein phosphatase 1, regulatory (inhibitor) subunit 1B | Ppp1r1b | 2,25 | 4,43E-03 |
| 1374776_at | BG380430 | vasohibin 2 | Vash2 | 2,23 | 3,69E-03 |
| 1371037_at | U06230 | protein S (alpha) | Pros1 | 2,23 | 2,08E-06 |
| 1384437_at | AI576309 | SWI/SNF related, matrix associated, actin dependent regulator of chromatin, subfamily a, member 1 | Smarca1 | 2,23 | 6,85E-04 |
| 1389651_at | AI177057 | apelin | Apln | 2,23 | 1,53E-03 |
| 1375278_at | BG670916 | tripartite motif-containing 2 | Trim2 | 2,23 | 1,66E-03 |
| 1374643_at | BF288461 | FAT tumor suppressor homolog 4 (Drosophila) | Fat4 | 2,23 | 6,95E-04 |
| 1370508_a_at | AF290212 | calcium channel, voltage-dependent, T type, alpha 1G subunit | Cacna1g | 2,23 | 3,55E-05 |
| 1387703_a_at | AF106659 | ubiquitin specific peptidase 2 | Usp2 | 2,23 | 1,40E-04 |
| 1387766_a_at | NM_012640 | retinol binding protein 2, cellular | Rbp2 | 2,23 | 7,22E-04 |
| 1388837_at | AI408306 | solute carrier family 44, member 2 | Slc44a2 | 2,22 | 2,02E-04 |
| 1387318_at | AY028605 | potassium large conductance calcium-activated channel, subfamily M, beta member 4 | Kcnmb4 | 2,22 | 2,12E-05 |
| 1380577_at | AI175616 | ATP-binding cassette, sub-family G (WHITE), member 2 | Abcg2 | 2,22 | 4,64E-06 |
| 1370186_at | AI599350 | proteasome (prosome, macropain) subunit, beta type 9 (large multifunctional peptidase 2) | Psmb9 | 2,22 | 2,53E-06 |
| 1381014_at | BI274623 | interferon-induced protein 44 | Ifi44 | 2,22 | 1,70E-05 |
| 1397304_at | AW525366 | interferon gamma induced GTPase | Igtp | 2,21 | 4,43E-05 |
| 1395512_at | AA866388 | cytokine receptor-like factor 1 | Crlf1 | 2,21 | 3,85E-04 |
| 1383247_a_at | BI291029 | spinster homolog 2 | Spns2 | 2,21 | 1,66E-04 |
| 1367616_at | NM_031545 | natriuretic peptide precursor B | Nppb | 2,20 | 1,44E-04 |
| 1387276_at | NM_021584 | doublecortin-like kinase 1 | Dclk1 | 2,20 | 1,21E-05 |
| 1379397_at | BE106199 | RAR-related orphan receptor A | Rora | 2,20 | 4,24E-05 |
| 1391534_at | BG666735 | elongation of very long chain fatty acids (FEN1/Elo2, SUR4/Elo3, yeast)-like 2 | Elovl2 | 2,19 | 1,82E-04 |
| 1391944_at | BI296237 | family with sequence similarity 184, member A /// similar to minichromosome maintenance protein 8 isoform 1 | Fam184a /// RGD1560557 | 2,19 | 1,77E-05 |
| 1398483_at | AI229118 | regulator of G-protein signaling 17 | Rgs17 | 2,19 | 9,22E-04 |
| 1383439_at | BI278550 | neuronal PAS domain protein 2 | Npas2 | 2,19 | 1,05E-03 |
| 1384381_at | BF284523 | ATP-binding cassette, sub-family A (ABC1), member 1 | Abca1 | 2,18 | 6,99E-04 |
| 1367838_at | NM_017074 | cystathionase (cystathionine gamma-lyase) | Cth | 2,18 | 1,70E-03 |
| 1374620_at | BM392373 | carcinoembryonic antigen-related cell adhesion molecule 1 (biliary glycoprotein) | Ceacam1 | 2,18 | 2,61E-06 |
| 1390789_at | BI296347 | acyl-Coenzyme A dehydrogenase family, member 11 | Acad11 | 2,18 | 3,99E-04 |
| 1387995_a_at | BI285494 | interferon induced transmembrane protein 3 | Ifitm3 | 2,18 | 4,34E-06 |
| 1372564_at | AI411375 | v-ets erythroblastosis virus E26 oncogene homolog 2 (avian) | Ets2 | 2,17 | 1,69E-04 |
| 1372604_at | BI289459 | similar to apolipoprotein L2; apolipoprotein L-II | RGD1309808 | 2,17 | 3,86E-04 |
| 1378239_at | BF404337 | glucosidase, alpha, acid | Gaa | 2,17 | 2,95E-05 |
| 1393626_at | BF562507 | sortilin-related receptor, LDLR class A repeats-containing | Sorl1 | 2,17 | 2,59E-03 |
| 1387850_at | NM_023020 | transmembrane protein with EGF-like and two follistatin-like domains 1 | Tmeff1 | 2,17 | 1,41E-05 |
| 1388060_at | U71294 | synaptotagmin XII | Syt12 | 2,17 | 1,70E-03 |
| 1377824_a_at | AI230709 | insulin-like growth factor 2 mRNA binding protein 3 | Igf2bp3 | 2,16 | 1,08E-02 |
| 1394501_at | BM390379 | sialic acid binding Ig-like lectin 10 | Siglec10 | 2,16 | 2,09E-03 |
| 1371799_at | AI175438 | glucosidase, alpha, acid | Gaa | 2,15 | 1,90E-04 |
| 1386794_at | BF544968 | F-box and WD-40 domain protein 7 /// F-box and WD-40 domain protein 7, archipelago homolog (Drosophila)-like | LOC100360914 /// LOC100365221 | 2,15 | 4,96E-03 |
| 1389403_at | AI013715 | bone morphogenetic protein 7 | Bmp7 | 2,15 | 3,53E-03 |
| 1388422_at | BI275904 | LIM and senescent cell antigen like domains 2 | Lims2 | 2,15 | 1,89E-03 |
| 1375211_at | BM391506 | ribonuclease T2 | Rnaset2 | 2,15 | 2,57E-06 |
| 1373233_at | AI013502 | lipoma HMGIC fusion partner-like 2 | Lhfpl2 | 2,15 | 9,62E-05 |
| 1377029_at | AI235414 | RAR-related orphan receptor alpha | Rora | 2,15 | 1,78E-04 |
| 1367986_at | NM_019243 | prostaglandin F2 receptor negative regulator | Ptgfrn | 2,15 | 3,78E-04 |
| 1377761_at | BI296057 | glutamine-fructose-6-phosphate transaminase 2 | Gfpt2 | 2,14 | 2,87E-04 |
| 1368543_at | NM_053524 | NADPH oxidase 4 | Nox4 | 2,14 | 1,10E-03 |
| 1387946_at | AF065438 | lectin, galactoside-binding, soluble, 3 binding protein | Lgals3bp | 2,14 | 1,17E-05 |
| 1369313_at | NM_031677 | four and a half LIM domains 2 | Fhl2 | 2,14 | 3,79E-04 |
| 1389706_at | AI007639 | doublecortin-like kinase 1 | Dclk1 | 2,14 | 2,38E-03 |
| 1371440_at | AW916647 | Beta-2 microglobulin | B2m | 2,14 | 1,54E-04 |
| 1391156_at | BI304040 | podocan-like 1 | Podnl1 | 2,14 | 1,37E-03 |
| 1370862_at | J02582 | apolipoprotein E | Apoe | 2,14 | 6,76E-05 |
| 1372955_at | BI282720 | serine/threonine kinase 38 like | Stk38l | 2,13 | 2,22E-03 |
| 1376151_a_at | AI407953 | rCG32755-like | LOC100365106 | 2,13 | 2,00E-05 |
| 1382072_at | AA944162 | olfactomedin-like 2A | Olfml2a | 2,13 | 1,06E-03 |
| 1384667_x_at | AI385327 | galanin receptor 2 | Galr2 | 2,13 | 3,43E-04 |
| 1398662_at | AA901088 | family with sequence similarity 167, member A | Fam167a | 2,13 | 6,36E-05 |
| 1376502_at | AW523504 | similar to putative protein, with at least 9 transmembrane domains, of eukaryotic origin (43.9 kD) (2G415) | RGD1309228 | 2,12 | 8,33E-05 |
| 1368438_at | NM_022236 | phosphodiesterase 10A | Pde10a | 2,12 | 3,36E-03 |
| 1376074_at | AA942690 | RAB11 family interacting protein 4 (class II) | Rab11fip4 | 2,12 | 5,75E-04 |
| 1383355_at | AW918387 | ATP-binding cassette, sub-family A (ABC1), member 1 | Abca1 | 2,12 | 4,98E-05 |
| 1379077_at | BE102740 | family with sequence similarity 181, member B | Fam181b | 2,11 | 7,43E-04 |
| 1377018_at | BF284124 | peptidase domain containing associated with muscle regeneration 1 | Pamr1 | 2,11 | 5,35E-03 |
| 1367577_at | NM_031970 | heat shock protein 1 | Hspb1 | 2,10 | 1,32E-05 |
| 1373577_at | BE116566 | Neuropilin 1 | Nrp1 | 2,10 | 3,41E-05 |
| 1370331_at | AF347936 | interleukin 11 receptor, alpha chain 1 | Il11ra1 | 2,09 | 1,47E-05 |
| 1371005_at | AI059506 | ATP-binding cassette, sub-family C (CFTR/MRP), member 1 | Abcc1 | 2,09 | 1,48E-04 |
| 1388145_at | BM390128 | tenascin XB | Tnxb | 2,08 | 1,04E-03 |
| 1393124_at | AI145807 | potassium voltage gated channel, shaker related subfamily, member 6 | Kcna6 | 2,08 | 7,69E-03 |
| 1377750_at | AI454536 | Rho guanine nucleotide exchange factor (GEF) 3 | Arhgef3 | 2,08 | 1,59E-04 |
| 1390107_at | BG670294 | synaptotagmin-like 2 | Sytl2 | 2,08 | 3,99E-04 |
| 1391656_at | AI101416 | carboxypeptidase M | Cpm | 2,08 | 2,39E-03 |
| 1382453_at | BE118707 | Zic family member 3 (odd-paired homolog, Drosophila) | Zic3 | 2,08 | 3,23E-04 |
| 1368363_at | NM_053394 | Kruppel-like factor 5 | Klf5 | 2,07 | 8,78E-05 |
| 1393510_at | AW524463 | Golgi-localized protein | Golsyn | 2,07 | 2,15E-03 |
| 1394039_at | BM382886 | Kruppel-like factor 5 | Klf5 | 2,06 | 1,97E-05 |
| 1376920_at | BF408536 | similar to sterile alpha motif domain containing 9-like | LOC500013 | 2,06 | 9,23E-04 |
| 1384187_at | BF551686 | adaptor-related protein complex 1, sigma 2 subunit | Ap1s2 | 2,06 | 4,08E-06 |
| 1371010_at | BF389361 | NK2 homeobox 1 | Nkx2-1 | 2,06 | 1,12E-04 |
| 1373992_at | AI408440 | similar to interferon-inducible GTPase | MGC108823 | 2,06 | 1,85E-05 |
| 1368059_at | NM_053955 | crystallin, mu | Crym | 2,06 | 5,81E-04 |
| 1387929_at | AB020504 | PMF32 protein | Pmf31 | 2,06 | 6,53E-04 |
| 1389166_at | BF403998 | calcium and integrin binding family member 2 | Cib2 | 2,05 | 1,74E-03 |
| 1389781_at | AI412625 | ELMO/CED-12 domain containing 1 | Elmod1 | 2,05 | 6,76E-03 |
| 1387184_at | NM_024355 | axin 2 | Axin2 | 2,05 | 1,25E-03 |
| 1368104_at | NM_022589 | tetraspanin 2 | Tspan2 | 2,05 | 5,45E-04 |
| 1387205_at | NM_022921 | RT1 class Ib, locus M3, gene 1 | RT1-M3-1 | 2,04 | 6,25E-05 |
| 1368563_at | NM_024399 | aspartoacylase | Aspa | 2,04 | 5,12E-03 |
| 1385014_at | BG377996 | Nudix (nucleoside diphosphate linked moiety X)-type motif 11 | Nudt11 | 2,04 | 9,43E-04 |
| 1382726_at | BF386091 | naked cuticle homolog 1 (Drosophila) | Nkd1 | 2,04 | 8,53E-04 |
| 1372691_at | BI292558 | uridine phosphorylase 1 | Upp1 | 2,03 | 4,61E-04 |
| 1383146_at | BM388077 | similar to neurobeachin | RGD1562629 | 2,03 | 4,50E-05 |
| 1373102_at | BI282750 | cadherin 13 | Cdh13 | 2,03 | 1,96E-04 |
| 1372729_at | AI137406 | protein C receptor, endothelial | Procr | 2,03 | 5,04E-05 |
| 1389307_at | AW435479 | amyloid beta (A4) precursor-like protein 1 | Aplp1 | 2,03 | 4,30E-03 |
| 1393901_at | BF408914 | runt-related transcription factor 1; translocated to, 1 (cyclin D-related) | Runx1t1 | 2,03 | 2,79E-03 |
| 1374872_at | AW532114 | RAS guanyl releasing protein 2 (calcium and DAG-regulated) | Rasgrp2 | 2,03 | 3,49E-03 |
| 1374883_at | BF392344 | myotubularin related protein 7 | Mtmr7 | 2,03 | 4,96E-04 |
| 1389246_at | BF282414 | UDP-Gal:betaGlcNAc beta 1,4- galactosyltransferase, polypeptide 1 | B4galt1 | 2,02 | 6,99E-06 |
| 1378536_at | AI638960 | hook homolog 1 (Drosophila) | Hook1 | 2,02 | 3,17E-04 |
| 1382181_at | AI555069 | pigeon homolog (Drosophila) | Pion | 2,02 | 9,47E-04 |
| 1381474_at | AI010322 | muscleblind-like 3 (Drosophila) | Mbnl3 | 2,02 | 1,54E-04 |
| 1373515_at | BI275737 | galectin-related protein | Hspc159 | 2,01 | 2,38E-05 |
| 1382590_at | BI277914 | raftlin lipid raft linker 1 | Rftn1 | 2,01 | 1,12E-04 |
| 1392012_at | BE100353 | similar to Epiplakin | LOC686567 | 2,01 | 1,33E-04 |
| 1367612_at | NM_134349 | microsomal glutathione S-transferase 1 | Mgst1 | 2,01 | 3,00E-05 |
| 1367960_at | NM_019186 | ADP-ribosylation factor-like 4A | Arl4a | 2,01 | 1,08E-04 |
| 1378699_at | AI176379 | polycystic kidney and hepatic disease 1-like 1 | Pkhd1l1 | 2,01 | 1,80E-05 |
| 1389913_at | BI276990 | leucine rich repeat (in FLII) interacting protein 1 | Lrrfip1 | 2,01 | 7,56E-06 |
| 1367701_at | NM_031646 | receptor (G protein-coupled) activity modifying protein 2 | Ramp2 | 2,01 | 7,77E-05 |
| 1390112_at | BF284634 | EGF-containing fibulin-like extracellular matrix protein 1 | Efemp1 | 2,01 | 2,74E-06 |
| 1374539_at | AA850290 | ATPase, class V, type 10D | Atp10d | 2,01 | 8,58E-05 |
| 1368266_at | NM_017134 | arginase, liver | Arg1 | 2,00 | 9,62E-06 |
| 1386269_x_at | AI177589 | sortilin-related receptor, LDLR class A repeats-containing | Sorl1 | 2,00 | 2,36E-02 |
