# Supplementary material for: The Histone H3K27 Methylation Mark Regulates Intestinal Epithelial Cell Density-Dependent Proliferation and the Inflammatory Response
Source: J Cell Biochem. 2012 Nov 28;114(5):1203–15. doi: 10.1002/jcb.24463 (PMC3617464; doi:10.1002/jcb.24463)
Supplement: Supplementary file 8 [file jcb0114-1203-SD8.doc]

**Supplementary Table 4.** Classification of genes induced more than 5 times in Suz12 depleted cells according to their GO biological process, using DAVID database.

| **Entrez Gene ID** | **Gene Name** | **Gene Symbol** | **Fold change** | **biological adhesion; GO:0022610 ; cell adhesion; GO:0007155** | **response to external stimulus; GO:0009605** | **regulation of multicellular organismal process; GO:0051239** | **response to wounding; GO:0009611** | **immune system process; GO:0002376** | **regulation of developmental process; GO:0050793** | **cell proliferation; GO:0008283** |
| --- | --- | --- | --- | --- | --- | --- | --- | --- | --- | --- |
| 4692 | necdin homolog (mouse) | Ndn | 64,53 |  |  |  |  |  |  | x |
| 3929 | lipopolysaccharide binding protein | Lbp | 38,44 |  | x | x | x | x |  |  |
| 2 | alpha-2-macroglobulin | A2m | 33,50 |  | x |  | x | x |  |  |
| 6696 | secreted phosphoprotein 1 | Spp1 | 31,57 | x | x | x | x |  | x |  |
| 81035 | collectin sub-family member 12 | Colec12 | 27,12 |  |  |  |  | x |  |  |
| 5270 | serpin peptidase inhibitor, clade E (nexin, plasminogen activator inhibitor type 1), member 2 | Serpine2 | 26,40 | x | x | x | x |  | x | x |
| 799 | calcitonin receptor | Calcr | 25,32 |  |  | x |  | x |  |  |
| 5274 | serpin peptidase inhibitor, clade I (neuroserpin), member 1 | Serpini1 | 23,64 | x |  |  |  |  |  |  |
| 11197 | WNT inhibitory factor 1 | Wif1 | 22,29 |  |  |  |  |  | x |  |
| 3597 | interleukin 13 receptor, alpha 1 | Il13ra1 | 20,43 |  |  |  |  | x |  | x |
| 3075 | complement factor H | Cfh | 19,95 |  | x |  | x | x |  |  |
| 1404 | hyaluronan and proteoglycan link protein 1 | Hapln1 | 19,16 | x |  |  |  |  |  |  |
| 3481 | insulin-like growth factor 2 (somatomedin A) | Igf2 | 18,80 |  |  |  |  |  |  | x |
| 55859 | brain expressed, X-linked 1 | Bex1 | 18,24 |  |  | x |  |  | x | x |
| 6347 | chemokine (C-C motif) ligand 2 | Ccl2 | 17,87 | x | x | x | x | x | x | x |
| 9843 | hephaestin | Heph | 17,39 |  |  |  |  | x |  |  |
| 133418 | embigin | Emb | 17,26 | x |  |  |  |  |  |  |
| 27330 | ribosomal protein S6 kinase, 90kDa, polypeptide 6 | Rps6ka6 | 16,49 |  | x |  |  |  |  |  |
| 1462 | versican | Vcan | 16,01 | x |  |  | x |  |  |  |
| 6304 | SATB homeobox 1 | Satb1 | 15,19 |  | x |  |  | x |  | x |
| 760 | carbonic anhydrase II | Car2 | 14,58 |  |  | x |  | x | x |  |
| 1501 | catenin (cadherin-associated protein), delta 2 (neural plakophilin-related arm-repeat protein) | Ctnnd2 | 14,10 | x |  | x |  |  |  |  |
| 93986 | forkhead box P2 | Foxp2 | 13,78 |  | x | x |  |  | x | x |
| 9289 | G protein-coupled receptor 56 | Gpr56 | 12,91 | x |  |  |  |  |  |  |
| 64094 | SPARC related modular calcium binding 2 | Smoc2 | 12,58 | x |  |  |  |  |  |  |
| 5101 | protocadherin 9 | Pcdh9 | 12,35 | x |  |  |  |  |  |  |
| 2331 | fibromodulin | Fmod | 12,07 |  |  |  | x |  |  |  |
| 3371 | tenascin C | Tnc | 11,86 | x |  |  | x |  |  | x |
| 3488 | insulin-like growth factor binding protein 5 | Igfbp5 | 11,74 |  |  | x |  |  | x | x |
| 91543 | radical S-adenosyl methionine domain containing 2 | Rsad2 | 11,60 |  |  | x |  | x |  |  |
| 10631 | periostin, osteoblast specific factor | Postn | 11,15 | x |  |  |  |  |  |  |
| 57161 | pellino homolog 2 (Drosophila) | Peli2 | 11,07 |  |  |  |  | x |  |  |
| 3827 | kininogen 1 | Kng1 | 11,07 | x | x | x | x | x |  | x |
| 10468 | follistatin | Fst | 11,03 |  |  | x |  | x | x | x |
| 6364 | chemokine (C-C motif) ligand 20 | Ccl20 | 10,59 |  | x |  | x | x |  |  |
| 10643 | insulin-like growth factor 2 mRNA binding protein 3 | Igf2bp3 | 8,67 |  |  | x |  |  |  |  |
| 139818 | dedicator of cytokinesis 11 | Dock11 | 8,66 |  |  |  | x |  |  |  |
| 7412 | vascular cell adhesion molecule 1 | Vcam1 | 8,56 | x | x |  | x | x |  | x |
| 2634 | guanylate binding protein 2, interferon-inducible | Gbp2 | 8,44 |  |  |  |  | x |  |  |
| 6423 | secreted frizzled-related protein 2 | Sfrp2 | 8,43 | x | x | x |  | x | x | x |
| 5168 | ectonucleotide pyrophosphatase/phosphodiesterase 2 | Enpp2 | 8,39 |  | x |  |  | x |  |  |
| 55106 | schlafen family member 12 | Slfn3 | 8,33 |  |  |  |  |  |  | x |
| 721 | complement component 4B (Chido blood group) | C4b | 8,21 |  |  |  | x | x |  |  |
| 4239 | microfibrillar-associated protein 4 | Mfap4 | 8,09 | x |  |  |  |  |  |  |
| 8092 | ALX homeobox 1 | Alx1 | 7,76 | x |  |  |  |  |  |  |
| 2770 | guanine nucleotide binding protein (G protein), alpha inhibiting activity polypeptide 1 | Gnai1 | 7,76 |  |  | x | x |  |  |  |
| 54829 | asporin | Aspn | 7,47 |  |  | x |  |  | x |  |
| 3627 | chemokine (C-X-C motif) ligand 10 | Cxcl10 | 7,42 |  | x | x | x | x | x | x |
| 57575 | protocadherin 10 | Pcdh10 | 7,40 | x |  |  |  |  |  |  |
| 4094 | v-maf musculoaponeurotic fibrosarcoma oncogene homolog (avian) | Maf | 7,18 |  |  | x |  |  | x |  |
| 2153 | coagulation factor V (proaccelerin, labile factor) | F5 | 7,13 | x |  |  | x |  |  |  |
| 23554 | tetraspanin 12 | Tspan12 | 7,10 |  |  | x |  |  | x |  |
| 7045 | transforming growth factor, beta-induced, 68kDa | Tgfbi | 7,08 | x |  |  |  |  |  | x |
| 5552 | serglycin | Srgn | 7,04 |  |  | x | x |  | x |  |
| 5308 | paired-like homeodomain 2 | Pitx2 | 6,99 |  |  |  |  |  |  | x |
| 2719 | glypican 3 | Gpc3 | 6,90 |  |  |  |  |  |  | x |
| 4291 | myeloid leukemia factor 1 | Mlf1 | 6,89 |  |  |  |  | x |  |  |
| 716 | complement component 1, s subcomponent | C1s | 6,86 |  |  |  |  | x |  |  |
| 10644 | insulin-like growth factor 2 mRNA binding protein 2 | Igf2bp2 | 6,41 |  |  | x |  |  |  |  |
| 25805 | BMP and activin membrane-bound inhibitor homolog (Xenopus laevis) | Bambi | 6,35 |  |  | x |  |  | x | x |
| 1400 | collapsin response mediator protein 1 | Crmp1 | 6,16 |  | x |  |  |  |  |  |
| 3606 | interleukin 18 (interferon-gamma-inducing factor) | Il18 | 6,13 | x | x | x |  | x | x | x |
| 8519 | interferon induced transmembrane protein 1 (9-27) | Ifitm1 | 5,93 |  |  |  |  | x |  | x |
| 4313 | matrix metallopeptidase 2 (gelatinase A, 72kDa gelatinase, 72kDa type IV collagenase) | Mmp2 | 5,89 | x | x | x | x |  |  |  |
| 2817 | glypican 1 | Gpc1 | 5,83 |  | x |  |  |  |  |  |
| 6474 | short stature homeobox 2 | Shox2 | 5,82 |  |  | x |  |  | x | x |
| 1902 | lysophosphatidic acid receptor 1 | Lpar1 | 5,76 |  | x | x |  |  | x |  |
| 214 | activated leukocyte cell adhesion molecule | Alcam | 5,63 | x | x |  |  |  |  |  |
| 650 | bone morphogenetic protein 2 | Bmp2 | 5,39 |  | x | x | x |  | x | x |
| 629 | complement factor B | Cfb | 5,39 |  |  |  |  | x |  | x |
| 6532 | solute carrier family 6 (neurotransmitter transporter, serotonin), member 4 | Slc6a4 | 5,38 |  |  | x |  |  | x | x |
| 2621 | growth arrest-specific 6 | Gas6 | 5,36 | x | x |  | x | x |  | x |
| 8321 | frizzled family receptor 1 | Fzd1 | 5,34 |  |  | x |  |  | x |  |
| 5320 | phospholipase A2, group IIA (platelets, synovial fluid) | Pla2g2a | 5,29 |  | x |  | x |  | x | x |
| 168667 | BMP binding endothelial regulator | Bmper | 5,28 |  |  |  |  |  |  | x |
| 7099 | toll-like receptor 4 | Tlr4 | 5,25 |  | x | x | x | x | x | x |
| 54510 | protocadherin 18 | Pcdh18 | 5,09 | x |  |  |  |  |  |  |
| 1278 | collagen, type I, alpha 2 | Col1a2 | 5,05 |  | x |  | x | x |  |  |
| 1281 | collagen, type III, alpha 1 | Col3a1 | 5,03 | x | x |  | x | x |  |  |
| 6352 | chemokine (C-C motif) ligand 5 | Ccl5 | 5,01 | x | x | x | x | x | x |  |
