# Supplementary material for: The Histone H3K27 Methylation Mark Regulates Intestinal Epithelial Cell Density-Dependent Proliferation and the Inflammatory Response
Source: J Cell Biochem. 2012 Nov 28;114(5):1203–15. doi: 10.1002/jcb.24463 (PMC3617464; doi:10.1002/jcb.24463)
Supplement: Supplementary file 9 [file jcb0114-1203-SD9.doc]

Supplementary Table 5. Status of H3K4 and H3K27 methylation of genes analysed in this study, in murine cells (Ku et al., 2008).

|  | **methylation on histone H3** | | | |
| --- | --- | --- | --- | --- |
| **Gene ID** | **H3K4** | **H3K27** | **H3K4/H3K27** | **None** |
| **Ccnd1** | x |  |  |  |
| **Cdkn1b** | x |  |  |  |
| **Cebpb** | x |  |  |  |
| **Cebpd** | x |  |  |  |
| **Ctnnb1** | x |  |  |  |
| **Cxcl2** | x |  |  |  |
| **Stat3** | x |  |  |  |
| **Cxcl10** |  | x |  |  |
| **Dusp8** |  | x |  |  |
| **Hoxb13** |  | x |  |  |
| **Ascl1** |  |  | x |  |
| **Calcr** |  |  | x |  |
| **Ccnd2** |  |  | x |  |
| **Cdkn1a** |  |  | x |  |
| **Cdkn1c** |  |  | x |  |
| **Cxcl1** |  |  | x |  |
| **Dkk2** |  |  | x |  |
| **Dusp2** |  |  | x |  |
| **Igfbp5** |  |  | x |  |
| **Ndn** |  |  | x |  |
| **Six3** |  |  | x |  |
| **Slc6a15** |  |  | x |  |
| **Ugt8** |  |  | x |  |
| **Wif1** |  |  | x |  |
| **Ccl2** |  |  |  | x |
| **Ccl20** |  |  |  | x |
| **Ccl5** |  |  |  | x |
| **Il6** |  |  |  | x |
| **Lbp** |  |  |  | x |
| **Lcn2** |  |  |  | x |
| **Slpi** |  |  |  | x |

Methylation on H3K4 and H3K27 described by Ku et al., 2008.
